# Generation of novel prebiotic oligosaccharide pools from fiber drives biological insight in bacterial glycan metabolism

**DOI:** 10.1128/aem.02077-24

**Published:** 2025-02-06

**Authors:** Chad Masarweh, Maria Maldonado-Gomez, Bruna Paviani, Mrittika Bhattacharya, Cheng-Yu Weng, Christopher Suarez, Shawn Ehlers-Cheang, Aaron Stacy, Juan Castillo, Nithya Krishnakumar, Karen A. Kalanetra, Daniela Barile, J. Bruce German, Carlito B. Lebrilla, David A. Mills

**Affiliations:** 1 Department of Food Science & Technology, University of California 117239 , Davis, California, USA; 2 Department of Chemistry, University of California 173250 , Davis, California, USA; Universita degli Studi di Napoli Federico II, Portici, Italy

**Keywords:** fiber, prebiotics, bifidobacteria, microbiome, synbiotics

## Abstract

Prebiotic oligosaccharides are dietary supplements that modulate the intestinal gut microbiome by selectively nourishing subsets of the microbial community with a goal to enhance host health. To date, the diversity of polysaccharide compositions in the fiber consumed by humans is not well represented by the limited scope of oligosaccharide compositions present in current commercial prebiotics. Recently, our UC Davis group developed a novel method to generate oligosaccharides from any polysaccharide fiber, termed Fenton’s Initiation Toward Defined Oligosaccharide Groups (FITDOG). Using this method, sugar beet pulp (SBP) was transformed into sugar beet oligosaccharides (SBOs) composed of arabinose- and galactose-containing oligosaccharides. Fecal fermentations of SBO and SBP produced similar shifts in donor-specific bacterial communities and acid metabolite profiles with a general enrichment of *Bacteroides* and *Bifidobacterium*. However, *in vitro* tests revealed more *Bifidobacterium* strains could consume SBO than sugar beet arabinan, and specific strains showed differential consumption of arabinofuranooligosaccharides or galactooligosaccharide (GOS) portions of the SBO pool. Genomic and glycomic comparisons suggest that previously characterized, arabinan-specific, extracellular arabinofuranosidases from *Bifidobacterium* are not necessary to metabolize the arabino-oligosaccharides within SBO. Synbiotic application of SBO with an SBO-consuming strain *Bifidobacterium longum* subsp. *longum* SC596 in serial fecal enrichments resulted in enhanced persistence among 9 of 10 donor feces. This work demonstrates a novel workflow whereby FITDOG creates novel oligosaccharide pools that can provide insight into how compositional differences in fiber drive differential gut fermentation behaviors as well as their downstream health impacts. Moreover, these oligosaccharides may be useful in new prebiotic and synbiotic applications.

**IMPORTANCE:**

Prebiotics seek to selectively alter the host microbiome composition or function, resulting in a concurrent health benefit to the host. However, commercial prebiotics represent a small fraction of the diversity of food polysaccharide compositions. In this work a novel method, Fenton’s Initiation Toward Defined Oligosaccharide Groups (FITDOG) was used to generate an oligosaccharide pool from sugar beet pulp (SBP). Sugar beet oligosaccharides (SBOs) resulted in similar changes to SBP in fecal enrichments; however, SBO could be consumed by more beneficial bifidobacterial strains than the cognate polysaccharide. These results demonstrate how the details of glycan structure have a profound influence on how gut bacteria metabolize food carbohydrates. The implications of this work are relevant to understanding how different dietary sources influence the human microbiome and extend to developing novel oligosaccharide pools for prebiotic applications.

## INTRODUCTION

Prebiotics and dietary fiber can enrich health-promoting microbial consortia in the gut (1, 2). Fermentable carbohydrates, on reaching the lower intestine, synergize with the gut microbiota, creating a unique environmental niche, and promote the production of desired microbial metabolites that benefit the host. Unfortunately, the reduction of dietary fiber in Western diets is increasingly linked to chronic disease (3), with both acute and chronic effects. Acutely, the loss of fermentable substrates leads inevitably to a loss in production of short-chain fatty acid (SCFA) metabolites responsible for feeding colonocytes, educating the immune system, and maintaining an anti-inflammatory tone (4). Chronically, lower intakes of fermentable carbohydrates lead to loss of saccharolytic capacity in the gut microbiome itself (5). An obvious solution is to restore complex carbohydrate to the human diet, but how? Efforts to address a dietary “fiber gap” are hampered by the general lack of a mechanistic understanding of how the gut microbiota, or individual species, consume large polysaccharides. Indeed, by comparison to the advances in high throughput (and user friendly!) tools for genomics and proteomics, comparable tools for glycomics are lacking. Lack of such methodologies hampers efforts to precisely define the basic glycan structures in foods, much less their catabolism by the gut bacteria, either as a consortium or by individual species.

Recently, Amicucci and coworkers (6) developed a process to produce oligosaccharides from polysaccharides to characterize large carbohydrates via a chemical cleavage reaction, termed “Fenton’s initiation toward defined oligosaccharide groups” (FITDOG), employing controlled, iron-mediated oxidation of glycosidic bonds to turn polysaccharides into a pool of constituent oligosaccharides compositionally representative of the parent polysaccharide. Combined with recently developed high-throughput monosaccharide (7) and linkage profiling (8), these methods can be used to describe the average primary structure of polysaccharides and oligosaccharides (9). In addition to characterizing polysaccharides, FITDOG is a scalable method of producing fermentable oligosaccharides that conserve the composition and glycosidic linkages of the original polysaccharide (10).

Current prebiotics are defined as “a substrate that is selectively utilized by host microorganisms conferring a health benefit” (11). Despite that rather broad definition, existing commercial prebiotics are simple oligosaccharides of limited complexity and do not represent the diversity of monomers present in the dietary fiber present in a range of foods. Given the expanding knowledge of fiber composition, driven in part by the Davis Food Glycopedia (12), one use of the FITDOG approach is to generate novel, soluble oligosaccharides that may possess prebiotic activity. Moreover, such an approach would enable the comparison of bacterial catabolism of a parent polysaccharide directly with the cognate oligosaccharides of the same polysaccharide. To explore this concept, we examined sugar beet polysaccharides as an initial test fiber. Sugar beet pulp (SBP) polysaccharides are composed mostly of arabinan, rhamnogalacturonan, galactan, and cellulose. At present, sugar beet pulp is the only commercialized source of purified arabinan. In this study, we examined sugar beet pulp-derived oligosaccharides generated using FITDOG, for their prebiotic potential by exploring their effect on fecal fermentations, their interactions with select bifidobacteria, and their suitability for synbiotic applications.

## MATERIALS AND METHODS

### Production of FITDOG oligosaccharides

Beet pulp pellets were subjected to the FITDOG protocol (6) and then purified at the pilot scale (10). Briefly, 6 kg of a 14% wt/vol water slurry of beet pulp pellets was continuously stirred into 35 L of 0.1 M H_2_SO_4_, pH 3, and 60°C. Then, 5.5 g of Fe_2_(SO_4_)_3_ ∙ 5H_2_O (adjusted to pH 3 with H_2_SO_4_) was added, and 10 L of 30% H_2_O_2_ was incrementally added while maintaining the suspension at 60°C for 2 hours. The reaction was allowed to come to room temperature, then ~8.7 L of chilled 1 M NaOH was added to bring the pH to 10 and was incubated for 1 hour. The reaction was neutralized with 800 mL 2 M HCl and was filtered with a 90–150 μm sieve.

Oligosaccharides were separated from particulates and large polysaccharides by microfiltration, followed by ultrafiltration, followed by nanofiltration, according to published protocols (13). Monosaccharides were removed by six discontinuous rounds of diafiltration, and desalination was conducted by electrodialysis (Magna Imperio Systems Desalination Unit – MIS Economy Benchtop System). The resulting solution of semi-purified sugar beet pulp FITDOG reaction products (referred to as sugar beet oligosaccharides [SBOs]) was freeze dried (HR7000-M, Harvest Right LLC, USA), blended in a coffee grinder, and stored in a vacuum desiccator.

### Oligosaccharide characterization and quantification

The carbohydrate content of the semi-purified FITDOG reaction was estimated with the anthrone method (14) using a L-arabinose for the concentration curve and the BioTek Synergy2 microplate monochrometer reading at 495 nm. The purified FITDOG reaction was estimated to be 58% carbohydrate, so a solution described as being “1% wt/vol SBO” contains 17.24 mg/mL purified FITDOG reaction and about 10 mg/mL beet pulp oligosaccharides. Monosaccharide analysis, linkage analysis, and linear α-(1,5)-arabinofuranooligosaccharide (5AOS) and linear β-(1,4)-D-galactopyranooligosaccharides (4GOS) composition analysis were performed as previously described (7, 8, 15). Briefly, for the monosaccharide analysis, samples were hard acid hydrolyzed, derivatized with 1-phenyl-3-methyl-5-pyrazolone, cleaned by chloroform-water extraction, and injected into an Agilent 1290 InfinityII UHPLC coupled to an Agilent 6495A QqQ/MS.

For linkage analysis, samples were permethylated, then hydrolyzed, derivatized, and injected into an Agilent 1290 InfinityII UHPLC coupled to an Agilent 6495A QqQ/MS. For 5AOS analysis, samples were reduced with NaBH_4_, purified, and injected into an Agilent 1290 InfinityII UHPLC coupled to an Agilent 6530 QTOF/MS. All three analyses were performed by comparing to a pool of monosaccharide, linkage, and multi-D.P. 5AOS, respectively, quality control standards. For monosaccharide analysis, external calibration curves were made for absolute quantitation, but a protocol for doing this with glycosidic linkage and 5AOS identity analysis has not yet been constructed.

### Bacterial strains and growth conditions

The following bacteria were used for pure cultures: *Bifidobacterium pseudocatenulatum* MP80, *Bifidobacterium pseudocatenulatum* SC585, *Bifidobacterium longum* subsp. *longum* (BLL) SC596, BLL SC215, BLL SC664, *Bifidobacterium longum* subsp. *infantis* ATCC 15697, *Bifidobacterium adolescentis* ATCC 15703, *Bifidobacterium animalis* subsp. *lactis* ATCC 27536, *Bifidobacterium catenulatum* subsp. *kashiwanohense* JCM15439, *Bifidobacterium bifidum* ATCC 29521, *Bifidobacterium bifidum* SC555, *Bifidobacterium breve* SC95, and *Limnosilactobacillus reuteri* DSM20016. All culturing was carried out in a Coy vinyl anaerobic bubble kept at 37°C with an atmosphere of 3% H_2_, ~5% CO_2_, and balance N_2_. Routine culturing was carried out with anaerobic Difco MRS + 0.05% L-cysteine HCl (MRSC), and carbohydrate-specific culturing was carried out with anaerobic modified MRS (mMRSC) (16). SBP and SBO were produced as described above. Sugar beet arabinan (SBA) was purchased from Neogen/Megazyme (Cat. No. P-ARAB).

### Growth curves

Growth curve cultures (200 µL) had a final inoculum and sugar concentration of 1%, were overlayed with 40 µL filtered mineral oil, and were incubated at 37°C in flat-bottomed, optically clear, 96-well, lidded plates in a BioTek Powerwave 2. The optical density (OD) readings were graphed with GraphPad Prism 9. To facilitate the comparison of the growth of *Bifidobacterium* strains, we implemented a metric to standardize observed OD and calculate comparable growth scores. The growth score of each culture is calculated by dividing a strain’s maximum OD by the best strain’s maximum OD and multiplying by 100. Consequently, growth scores, expressed in percentage, represent how well a strain grew relative to the maximum growth recorded (which was typically BLL SC596). The rationale behind using BLL SC596 as the best grower to calculate scores is based on both maximum OD and glycan depletion. Prior to collecting data, discrete growth characterizations (good, moderate, poor, and no growth) were assigned to arbitrary ranges of growth scores as follows: no growth 0%–20%, poor growth 21%–35%, moderate growth 36%–60%, and good growth 61%–100%.

### Simulated upper intestinal digestion

*In vitro* digestion of sugar beet pulp was performed with the INFOGEST 2.0 protocol (17). Sugar beet pulp pellets were hydrated and then mixed with an equal volume of simulated salivary fluid (the final concentration of salivary amylase [Sigma A6814] was 75 IU/mL). During the simulated gastric phase, the final concentration of pepsin (Sigma, P6887) was 2,000 U/mL. No gastric lipase was added. During the simulated intestinal phase, the final concentration of pancreatin from porcine pancreas (Sigma, P7545) was 100 U/mL, and that of bile extract (Sigma, B3883) was 10 mmol/mL. The pancreatin enzymes had to be sonicated to be solubilized before addition to the digestion. The final digested product was immediately frozen at −20°C to halt enzymatic activity, before dialysis in 2000 NMWCO dialysis tubes (Sigma, D7884) for 4 days at 4°C against deionized water. The digested and dialyzed product was freeze dried (Harvest Right, HR7000-M), then blended with a coffee grinder and stored in a room temperature vacuum desiccator. The freeze-dried product is referred to as sugar beet pulp (SBP).

### Comparative genomics

Orthology searches of *Bifidobacterium* genomes were conducted within JGI’s IMG platform. dbCAN3 (18) was used to annotate the glycoside hydrolase content of *Bifidobacterium* genomes. CAGECAT was used to construct the synteny graph of *Bifidobacterium* genomes (19).

### Fecal fermentations

Two independent experimental designs were carried out using fecal fermentations: batch fecal fermentations using SBO and SBP inputs, and “synbiotic” serial fecal fermentations examining the persistence of BLL SC596 in combination with SBO. All fermentations were conducted in triplicate from fecal donors. All procedures involving the use of human fecal samples were approved by The University of California Davis Institutional Review Board (IRB #1600677-6). A total of 10 donor feces were used for the SBP and SBO fecal fermentations, and a modified set of 10 was used for the “synbiotic” serial fecal fermentations. Choice of fecal samples to use was based solely on fecal availability from the 20 donor set (wherein each was ascribed a simple number [i.e., donor 1–20]). Donors 2, 3, and 10 were used solely in the initial batch fecal fermentations; donors 20, 18, and 15 were used solely in the “synbiotic” serial fecal enrichment experiment; and donors 4, 5, 7, 8, 11, 13, and 19 were used in both.

To prepare the fecal inoculum, frozen feces were thawed in the anaerobic chamber at room temperature, and 14.4 g was weighed into a 50 mL centrifuge tube containing 21.6 mL of sterile, de-oxygenated 0.67× PBS, 33.33% vol/vol glycerol, then vortexed at full speed for 5 minutes. The slurry was centrifuged for 5 minutes at 200 × *g* to settle non-microbial solids. In the anaerobic chamber, 12.5 mL of the supernatant was transferred into each of seven 15 mL centrifuge tubes and was frozen at −80°C until needed.

The fermentation medium composition was based on that of Walker and coworkers (20), and was composed by combining the following components per liter DI water: biotin, 100 μg; CaCl_2_ • 2H_2_O, 20 mg; FeSO_4_ • 7H_2_O, 5.4 mg; L-Cysteine HCl, 500 mg; bile salts, 50 mg; Bacto Casitone, 3 g; Bacto Proteose peptone No. 3, 3 g; NaCl, 2.5 g; MgSO_4_ • 7H_2_O, 500 mg; hemin, 0.1; K_2_HPO_4_, 5 g; KH_2_PO_4_, 3.19 g; NaHCO_3_, 1.35 g; Na_2_CO_3_, 1.63 g; Tween-80, 2.12 g; and MES • H_2_O, 9.76 g; xylan from corn core, 80 mg; amylopectin from maize, 80 mg; citrus pectin, 80 mg; larch tree arabinogalactan, 80 mg; cellobiose, 5 mg; and potato starch, 675 mg; EDTA, 1 mg; ZnSO_4_ • 7H_2_O, 20 μg; MnCl_2_ 7H_2_O, 6 μg; boric acid, 60 μg; CoCl_2_ • 6H_2_O, 40 μg; CuCl_2_ • 2H_2_O, 2 μg; NiCl_2_ • 6H_2_O, 4 μg; NaMoO_4_ • 2H_2_O, 6 μg; menadione, 1 μg; para-aminobenzoic acid, 0.5 μg; pantothenate, 10 μg; nicotinamide, 5 μg; cyanocobalamin, 0.5 μg; and thiamine-HCl, 4 mg. Stock solutions were filter sterilized with a 0.22 μm polyethersulfone filter, and the background polysaccharide solution was autoclaved.

SBO and autoclaved SBP were fermented at a final carbohydrate concentration of 10 mg/mL. SBP was assumed to be 100% carbohydrate for the sake of simplicity. Fermentation tubes were held in a custom-made rack on top of a 60-magnet magnetic stir plate (MIXdrive 60, basic version, from 2mag-USA) and were homogenized with 8 × 3 mm stir bars stirring at full speed (1,200 rpm).

Fecal inocula were thawed in ice in the anaerobic chamber, then a volume of 6.67% of the fermentation medium volume was added to the fermentation tubes. Samples were collected at 0, 10, 24, and 48 hours after the first inoculation for pH, short-chain fatty acid analysis, and DNA extraction. Samples for DNA were directly transferred into BashingBeads tubes (Zymo Research, S6012) containing 1 mL 1× DNA shield (Zymo Research, R1100), briefly vortexed, and stored at room temperature until DNA extraction days later.

### Serial batch fermentations

The feces fermentation protocol above was replicated, except that after the initial 24 hours, sequential cultures were inoculated from the previous one via a 1% vol/vol transfer every 24 hours up to 96 hours of total fermentation (see method illustrated in Fig. 8A ). Additionally, BLL SC596 was added at a final concentration of 1% vol/vol to half the fermentations, with the other half being control fermentations without the strain. The inoculum was prepared by culturing the strain overnight in mMRSC + 1% SBO. The CFU per milliliter of the strain was interpolated via qPCR with strain-specific primers, where the standard curve template DNA was derived from a CFU per milliliter count of SC596 serially diluted in triplicate and plated in duplicate on MRS agar.

### DNA extraction, 16s rRNA gene sequencing, and qPCR

Fecal fermentation samples were disrupted by bead-beating in a FastPrep-24 (MP, 116004500) for 1 minute at 6.5 m/s, then incubated on ice for 5 minutes. This was repeated four more times. Lysed samples were centrifuged at 10,000 × *g* for 1 minute, and 200 µL of the supernatant was aliquoted into a KingFisher 96 Deep Well plate (ThermoFisher, 95040450). DNA extraction proceeded in a KingFisher Flex Robot (ThermoFisher, 5400630) with the ZymoBIOMICS 96 MagBead DNA Kit (Zymo Research, D4306) according to the kit protocol. Library preparation was started as described previously (21) except that PCRs were performed in triplicate, and the all triplicates of all samples were pooled before completing library preparation. Library preparation was completed, and the amplicon pool was sequenced by the UC Davis DNA Technologies Core with a MiSeq PE250.

In Qiime2 v2022.11 (22), sequences were demultiplexed with Sabre, quality filtered with DADA2, and feature filtered with Qiime2, and amplicon sequence variants (ASVs) were classified by sklearn with Silva non-redundant SSU reference database 138, 99%. Each sample was rarefied at 14,000 read pairs, and dissimilarity was calculated in Qiime2. Dissimilarity comparisons were conducted in R v4.2.2 with the following vegan (23) v2.6-4 functions: permutest.betadisper to compare dissimilarity dispersion, adonis2 to assess dissimilarity difference, metaMDS to ordinate the dissimilarity matrix, and the package ggplot2 v3.4.1 (24) to visualize the non-metric multidimensional scaling (NMDS). GraphPad Prism 9 was used to construct stacked bar plots of ASV relative abundance.

QPCR primers specific for BLL SC596 were designed to target a gene encoded by that strain but not encoded by other *Bifidobacterium* strains tested in this work’s growth curves. Candidate genes were found in the JGI/IMG platform. Primers targeting the identified regions were found with Primer3 v.4.1.0 (25), and candidate amplicons that had BLAST hits were removed from consideration. A 74 bp region of locus tag BLNG_01612 (JGI IMG database) was the final target. With 0.5 mM forward primer (5′-AGAAGTCCCGCAGCTTTGAC-3′) and 0.5 mM reverse primer (5′-TGGTGACGGATGGCATTCC-3′), 3 μL of template DNAs diluted 1:10 was amplified with PowerUp SYBR Green 2x Master Mix in an Applied Biosystems 7500 Fast in fast mode with amplification conditions as follows: 50°C for 2 minutes, 95°C for 2 minutes; 40 cycles of 95C° for 3 minutes then 60°C for 30 seconds.

### Metabolite quantification

SCFA analysis was performed as previously described (26). Briefly, 10 µL of the standard and sample was derivatized by adding 200 µL of acetonitrile and 100µL of 20mM triphenylphosphine, 20mM dipyridyl disulfide, and 20mM 2-picolylamine, and incubating at 60°C for 10 minutes. The reaction was desiccated with a miVac concentrator and was reconstituted in 50% methanol. The derivatized standards and samples were analyzed on an Agilent 6495B QqQ MS coupled with an Agilent 1290 Infinity II UHPLC. Separation was performed on an Agilent Poroshell 120 EC-C18 column (2.1 × 100 mm, 1.9 µm particle size). An external calibration curve was used for absolute quantitation.

## RESULTS

### Generation and characterization of sugar beet oligosaccharides

**Fig 1:**
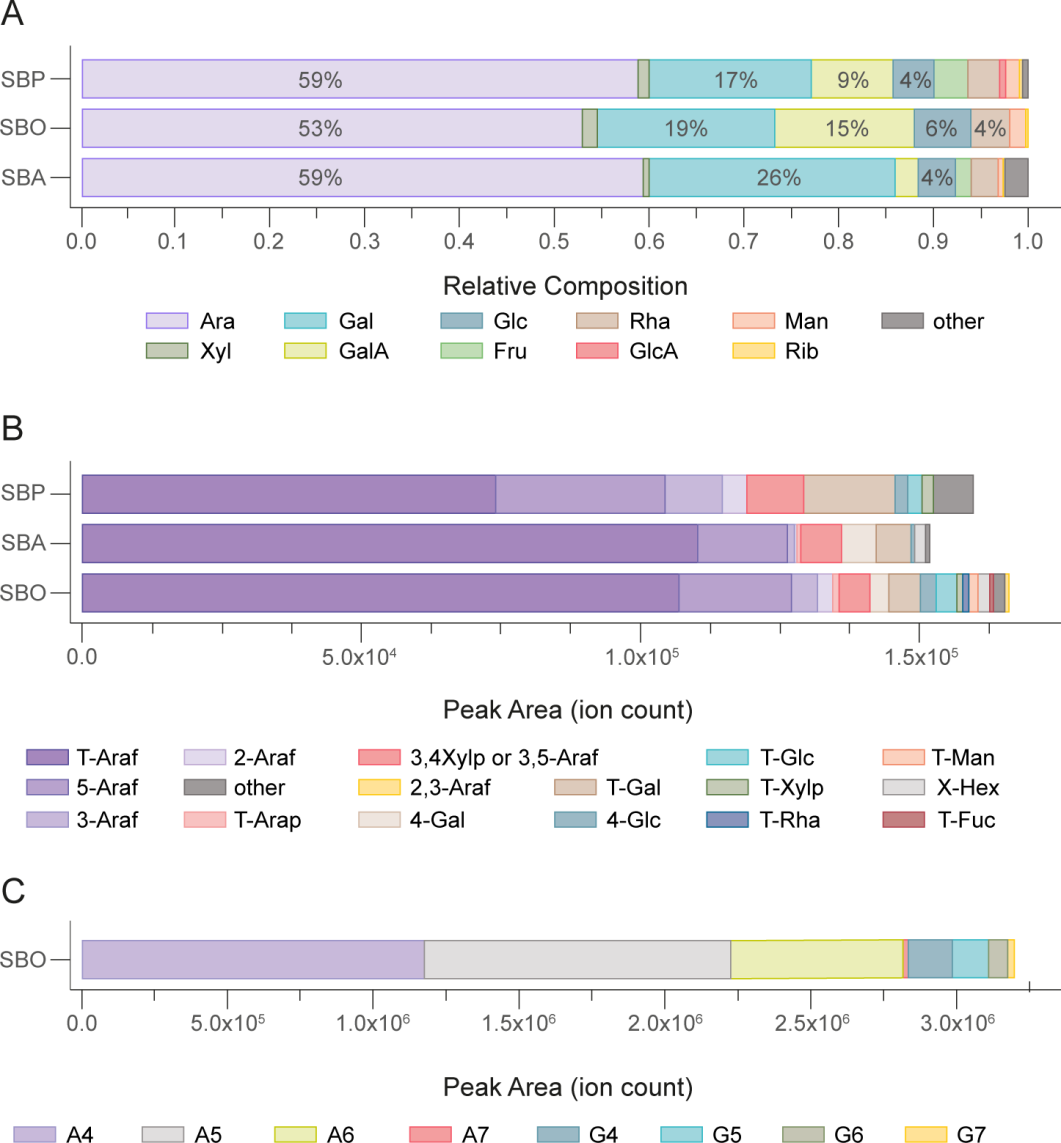
Carbohydrate composition of SBP, SBO and SBA in terms of (**A**) relative abundance of total monosaccharides, (**B**) integrated peak area of glycosidic linkages, and for SBO (C) integrated peak area of linear α-(1,5)-arabinofuranooligosaccharides (abbreviated “A” plus the degree of polymerization) and linear β-(1,4)-galactopyranooligosaccharides (abbreviated “G” plus the degree of polymerization).

An overarching goal of this work was to examine how oligosaccharides generated from parent polysaccharides are able to provide insights into both oligo- and polysaccharide degradation by gut microbiome. To pilot this approach, we examined sugar beet pulp, a byproduct of refined sugar production, which is currently added to livestock feed. Subjecting SBP to the FITDOG process and its subsequent pilot-scale purification yielded a mixture of SBOs that are soluble with similar compositions to the parent polysaccharides (10). The carbohydrate structure of SBO was characterized for total monosaccharide and glycosidic linkages (Fig. 1). The SBO was also profiled using liquid chromatography–mass spectrometry (LC-MS) to characterize the oligosaccharide products of the FITDOG process. Monosaccharide and linkage analysis was also performed on the parent SBP as well as the commercially-treated SBA. The relative abundances of monosaccharides in SBP, SBA, and SBO were generally similar (Fig. 1A). The SBO preparation contained more galacturonic acid than SBA because the manufacturer of SBA treated their input with galacturonidase before purification. Although arabinose is the major component of all three, there is a significant fraction of galactose and galacturonic acid in SBO and SBP. The SBO produced from the SBP, therefore, likely contains oligosaccharides that can potentially be analogous to galactooligosaccharides (GOS), rhamnogalacturonooligosaccharides, and/or arabinooligosaccharides, and yet their structures remain bound at their non-reducing end to rhamnogalacturonooligosaccharides. The linkage analysis yielded similar variations due to the strong similarities between the three sets of material (Fig. 1B). Close inspection will show that the sum of the linkage abundances may not specifically match those of the monosaccharide. The monosaccharide compositions are determined using standards. No such standards exist for linkage analysis, so that variations in the acid hydrolysis, methylation reaction, and ionization efficiencies will produce somewhat diverging MS responses. Nonetheless, fold changes of specific linkages are still precise, while comparison of different linkages may be less so.

### SBO and SBP induce mostly donor-dependent responses and a prevalent increase in *Bifidobacterium* and *Bacteroides*

To compare the impact on the microbiota, we examined human fecal fermentations containing SBO, or the parent SBP. SBOs were obtained as described previously (10). SBP was processed via the standard human digestion protocol (27). Samples were taken at 0, 10, 24 and 48 hours of fermentation, and changes of bacterial community composition were followed by 16S rRNA gene amplicon sequencing. To determine if differences between microbial communities were significant, permutational analysis of variance (PERMANOVA) was applied to beta-diversity metrics via the adonis2 function in the R package vegan.

**Fig 2:**
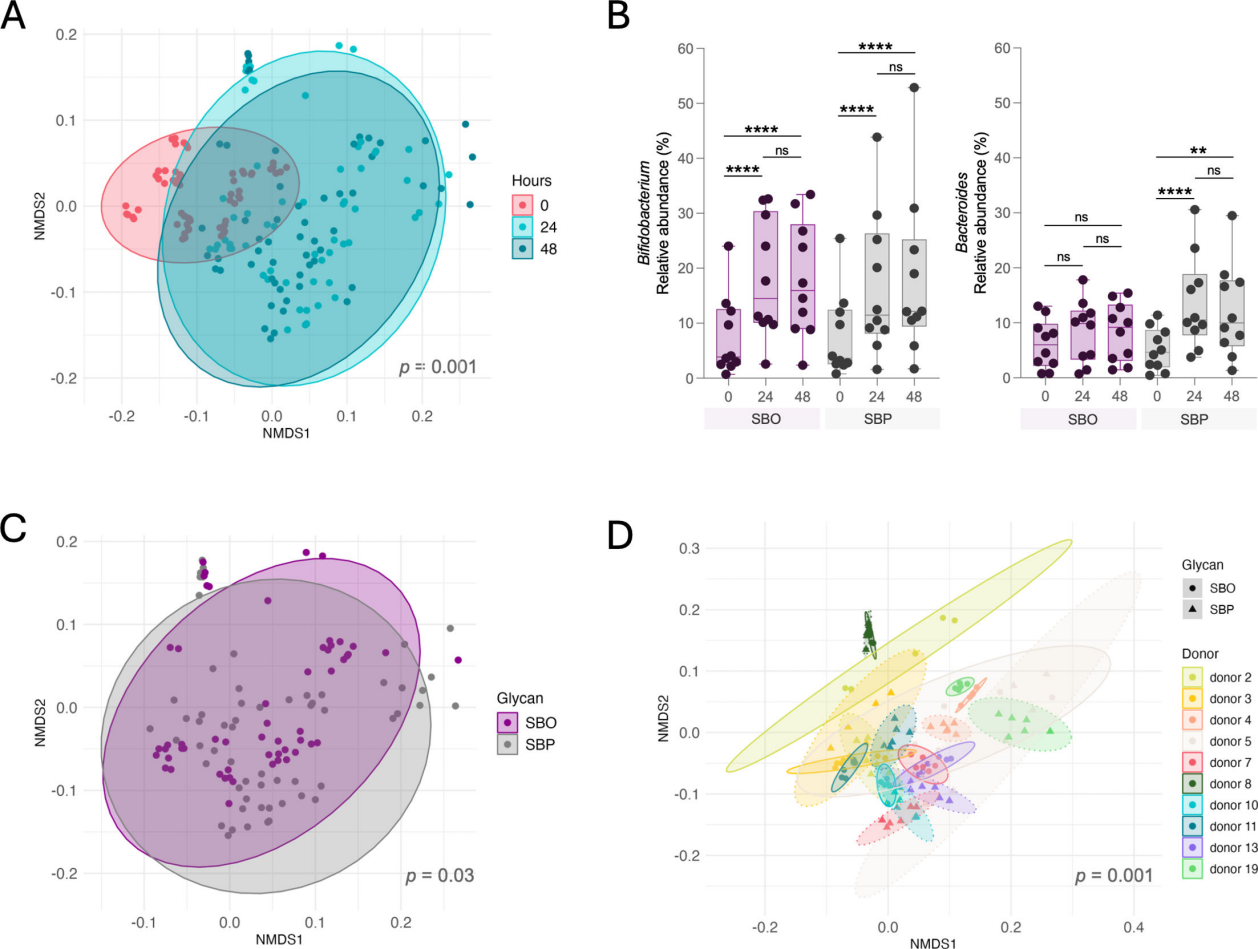
SBO and SBP induce mostly donor-dependent responses and a prevalent increase in *Bifidobacterium* and *Bacteroides*. (**A**) NMDS clustering of communities at different time points of the fecal fermentations, including all donors and all treatments. (**B**) Mean relative abundance of members belonging to *Bifidobacterium* or *Bacteroides*, as determined by 16S sequencing. Differences between groups were determined using a mixed-effects model with the Geisser-Greenhouse correction and Tukey’s multiple comparisons test. **P < 0.01, ****P < 0.0001. (**C**) NMDS clustering of communities derived from fecal fermentations supplemented with either SBO or SBP includes communities characterized at 24 and 48 hours. (**D**) NMDS clustering of fecal fermentation communities colored by donor. Shapes denote whether fermentations received SBO or SBP. Communities’ dissimilarities for panels (**A**), (**C**), and (**D**) were calculated using weighted UniFrac.

As expected, starting microbial communities were significantly different between donors (BH-adjusted *P* < 0.001). Regardless, both the oligosaccharide (SBO) and polysaccharide (SBP) produced shifts in fecal communities away from the starting inocula. Together, 24- and 48-hour-old communities were significantly dissimilar from those at 0 hour (Fig. 2A; Table S1). However, few ASVs change among most of the population (Fig. S1). Genera that consistently decreased in relative abundance in all or most of the fermentations were *Blautia* and *Faecalibacterium*, respectively. Conversely, *Bifidobacterium* and *Bacteroides* increased in all fermentations except for two donors (Fig. 2B).

When investigating the effects of the carbohydrate treatments on the overall structure of the community, differences between SBO and SBP post-fermentation reach significance depending on the dissimilarity index used to evaluate beta-diversity. While Bray-Curtis (BC) and unweighted UniFrac (UWU) suggest that SBO communities are not significantly different from those resulting from SBP supplementation (BH-adjusted BC *P* = 0.083, BH-adjusted UWU *P* = 0.122), weighted Unifrac indicates communities growing on SBO and SBP are in fact significantly different (BH-adjusted *P* = 0.03) (Fig. 2C). However, it was not a substantial difference (pseudo-F [effect size] = 2.401; *R*^2^ [fraction variation explained] = 0.013). This suggests that SBO and SBP have the potential to consistently enrich their own unique communities, but the small donor sample size (*n* = 10) and the large inter-individual community variation overpower the effect of carbohydrate supplementation.

Within most donors, the resulting bacterial communities by 24 and 48 hours significantly differed between SBO and SBP (Fig. 2D; Table S2) with donor 5 an exception (BH-adjusted *P* > 0.05). Substrate supplementation explains over 70% of the observed community dissimilarities (i.e., *R*^2^) in 3 of the 10 tested donors (4, 10, and 11) and over 20% for four additional donors (2, 5, 8, and 13). Adonis analysis of SBO and SBP samples, respectively, from donors 3, 7, and 19 could not be interpreted because their beta dispersions by carbohydrate were significantly different (Table S2). More replicates are required for a better analysis of those donors. In summary, differences between SBO- and SBP-enriched fecal communities could only be inferred within each donor but not between donors.

### Decrease in pH occurs faster in SBO fermentations and results in larger levels of selected SCFA

**Fig 3:**
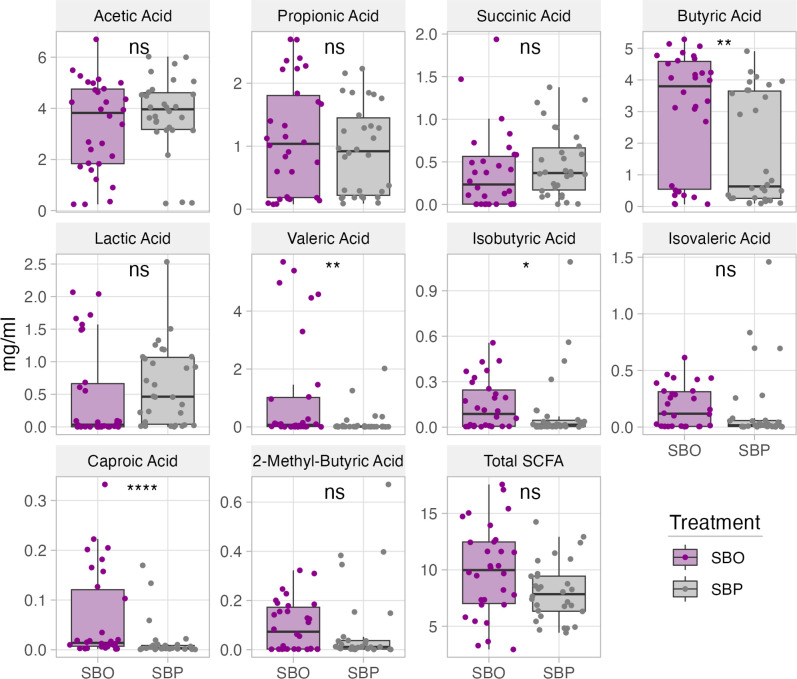
Comparison of the average concentrations of total and individual SCFAs in fecal fermentations supplemented with SBO or SBP. The figure depicts SCFA measurements from samples collected after 48 hours of fecal fermentation, conducted in triplicate for 10 different donors. Wilcoxon test was used to evaluate differences between groups. *P < 0.05, **P < 0.01, ***P < 0.001.

We explored SBO and SBP fermentability (via pH) and their effect on the resulting SCFA profile, as the latter is the primary end-product of non-digestible carbohydrates fecal fermentations. Notably, the pH is lower in SBO fermentations after 10 and 24 hours (Wilcoxon test, *P* = 0.018 and 0.04, correspondingly); however, by 48 hours, the pH was equivalent for both substrates (*P* = 0.65) (Fig. S2A) Consistently, at 48 hours, the total SCFA concentrations were not significantly different between SBO and SBP fermentations (Wilcoxon test, *P* = 0.37) (Fig. 3). Furthermore, the total concentration of SCFA was not correlated with pH in either SBO fermentations (Pearson’s, *P* = 0.43) or SBP fermentations (Pearson’s, *P* = 0.31). Butyric, valeric, isobutyric, and caproic acids were significantly more concentrated in the SBO than in the SBP fermentations (Fig. 3). The final pH of SBO fermentations was weakly correlated with the concentration of acetic (Pearson’s, *P* = 0.017, *r* = −0.43), lactic (Pearson’s, *P* = 0.0078, *r* = −0.48), and succinic (Pearson’s, *P* = 0.045, *r* = −0.37) acids, but the final pH of SBP fermentations was not correlated with the concentration of any measured acids (Table S3). It is important to consider that the fermentation medium has a strong buffer capacity, which influences pH readings, masking some of these differences. Another consideration is that SCFAs were measured at 48 hours of fermentation and key enriched taxa, such as bifidobacteria, produce metabolites (e. g. lactate and acetate) that are likely consumed by other taxa within the trophic network. Future studies would benefit from sampling at multiple time points throughout fermentation to capture changes in the primary metabolic products of bifidobacteria and provide further insights into metabolic networks.

### *Bifidobacterium* consumption and oligosaccharide preference for SBO are species and strain specific

**Fig 4:**
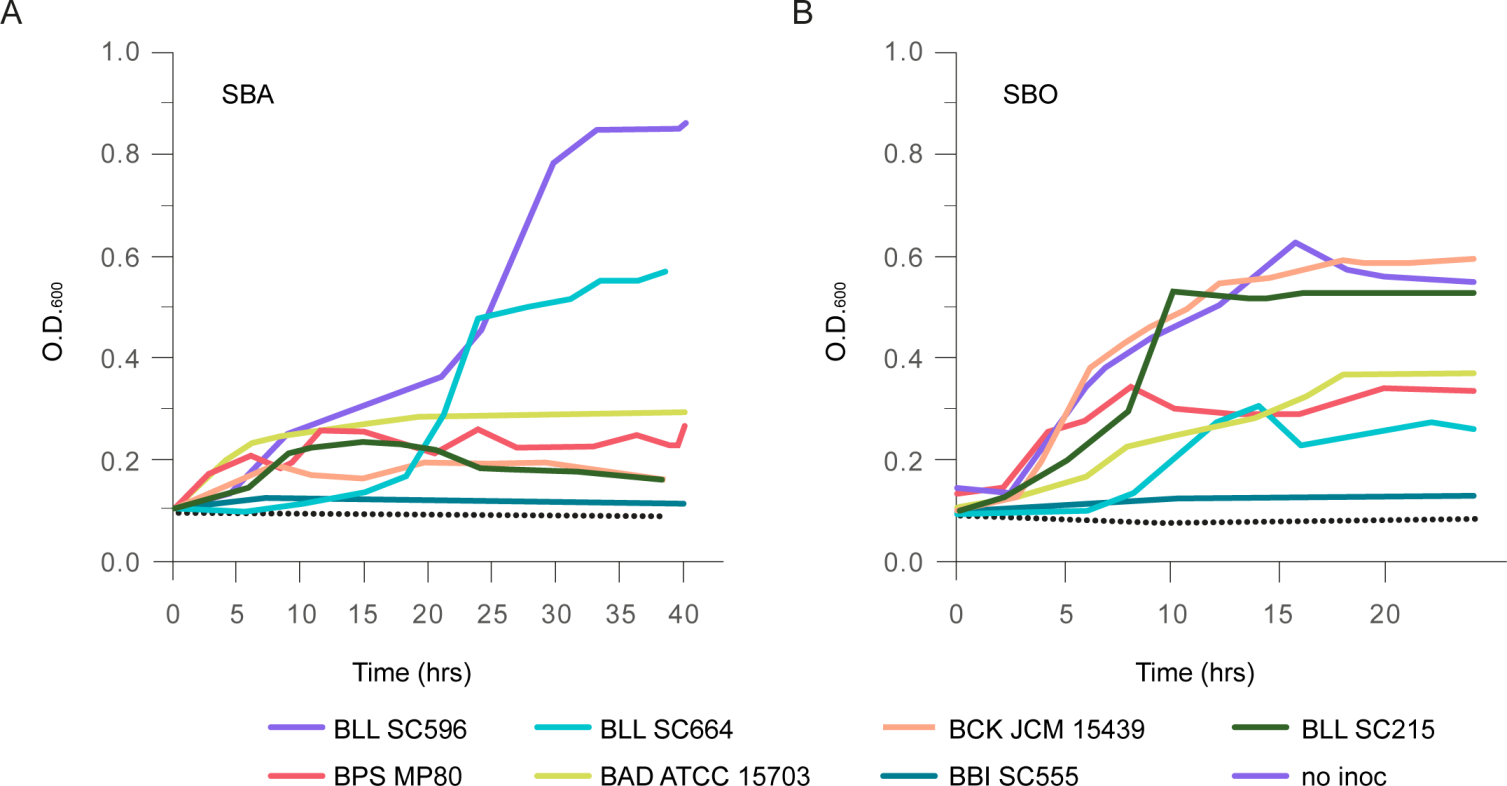
Optical density of *Bifidobacterium* cultures plotted over time. Strains were cultured in (**A**) SBA and (**B**) SBO as a sole carbon source. All strains were cultured in duplicate in mMRSC + 1% carbohydrate. Species names are abbreviated as follows: *B. longum* subsp. *longum* (BLL), *B. catenulatum* subsp. *kashiwanohense* (BCK), *B. adolescentis* (BAD), *B. pseudocatenulatum* (BPS), and *B. bifidum* (BBI).

Because sugar beet carbohydrates enriched *Bifidobacterium* in this study’s *in vitro* feces fermentations and those of others, we examined the ability of representatives of human gut *Bifidobacterium* species to degrade and metabolize SBO and SBA as a sole carbon source in pure culture. In this case, soluble SBA was used instead of SBP, as SBA has a similar monosaccharide composition to that of SBP (Fig. 1), and SBP is insoluble. SBA has been previously reported to be bifidogenic (28). In this work, of seven species tested, only BLL SC596, and to a lesser extent BLL SC664, showed good growth on SBA ( Fig. 4A; Table S4), while BLL SC215, *B. pseudocatenulatum* (BPS) MP80, and *B. adolescentis* (BAD) ATCC 15703 exhibited poor growth, and no growth was observed by *B. bifidum* (BBI) SC555. Growth of the same seven strains on SBO showed a different result. Three strains, BLL SC596, BLL SC215, and BCK JCM 15439 grew well on SBO, while moderate growth was observed from BPS MP80, BAD ATCC 15703, and BLL SC664, and no growth was observed with BBI SC555 ( Fig. 4B; Table S4). Screening of additional bifidobacterial strains for growth on SBO revealed one more strain, BPS SC585, which grew well; three strains, *Bifidobacterium breve* (BBR) SC95, *B. animalis* subsp. *lactis* (BAL) ATCC 27536, and *B. longum* subsp. *infantis* ATCC 15697 showed moderate growth, and BBI ATCC 29521 exhibited no growth (Fig. S3). That neither BBI strains grew on SBO is possibly due to the known limitation of the species *B. bifidum* to grow on arabinose (29). Finally, the lag time for growth of BLL SC596 and BLL SC664 was notably longer than that for SBO, perhaps due to the expected delay in catabolism of the larger arabinan by comparison with the smaller oligosaccharide pool.

**Fig 5:**
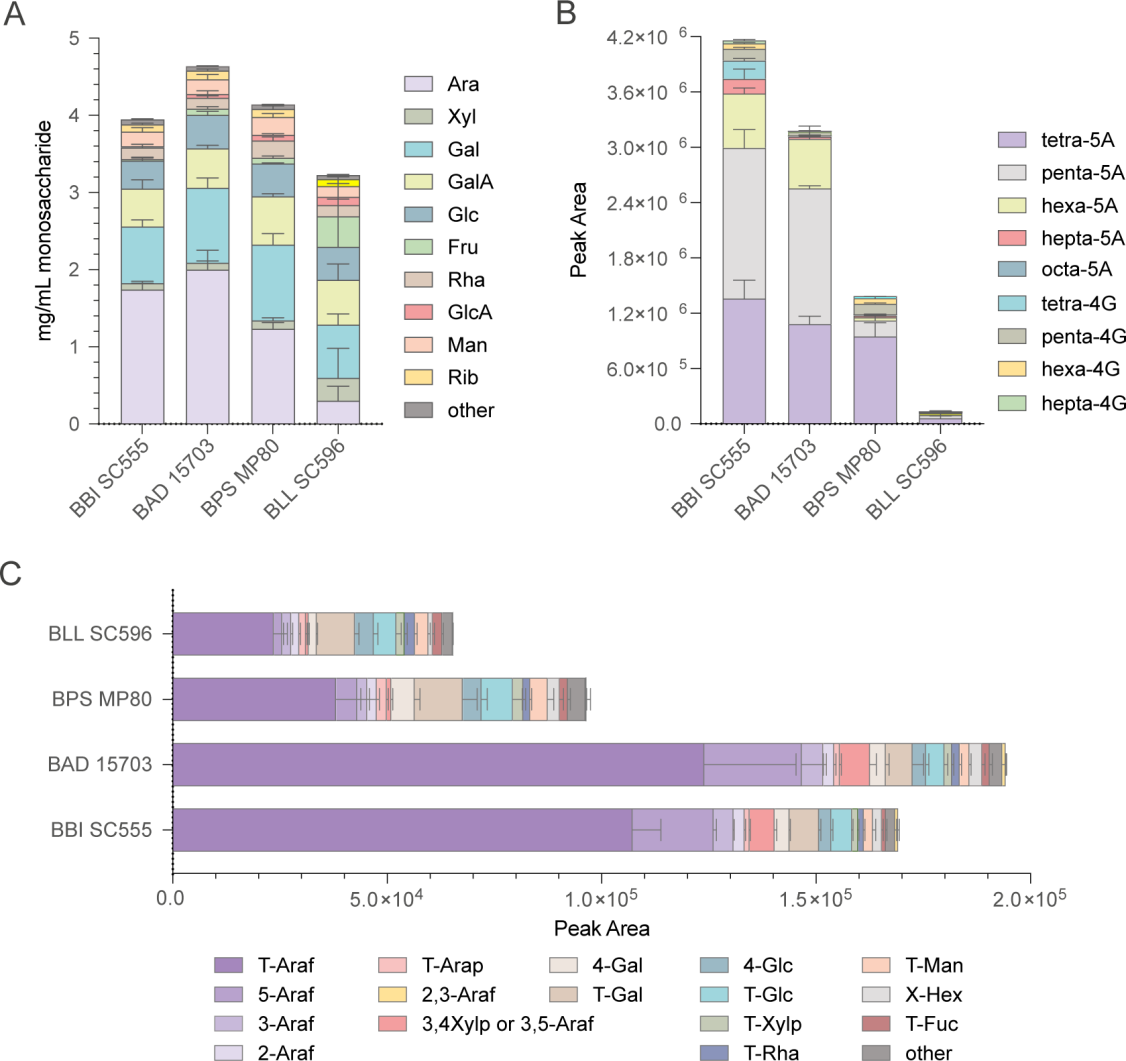
Oligosaccharide consumption of *Bifidobacterium* strains in pure culture. Stacked bar plots of (**A**) the concentration of total monosaccharides and of (**B**) the integrated peak area of product ions representing α-(1,5)-arabinofuranooligosaccharides and β-(1,4)-galactopyranooligosaccharides and of (**C**) measured glycosidic linkages in the spent media of 24 hour pure-culture, growth curve fermentations of mMRSC + 1% SBO. Each strain was grown in triplicate. Statistical differences can be found in Table S4.

To understand what components of the SBO pool were being consumed, we employed monosaccharide, glycosidic linkage, and oligosaccharide analysis on select SBO cultures. Supernatants of BBI SC555 are used as a control given that its culture showed no change in OD_600_. Using an inoculated culture accounts for minimal metabolism and other baseline changes caused by added cells. Consistent with the strong growth of BLL SC596 on SBO (Fig. 4B), supernatants of this culture exhibited the lowest concentration of arabinose (Fig. 5A), the smallest peak area of all measured 5AOS and 4GOS oligosaccharides (Fig. 5B), and the smallest peak area of arabinosyl linkages (Fig. 5C; Table S5). In fact, the peak area of all arabinosyl linkages (except T-Ara*p* and 2,3-Ara*f*) from BLL SC596 SBO fermentations (Fig. 5C; Table S5) was substantially smaller than in the control, suggesting BLL SC596 degraded most linear and branched arabinooligosaccharides.

While both BPS MP80 and BAL ATCC 15703 reached roughly equivalent final optical densities during growth on SBO, linkage and oligosaccharide profiles suggested differential consumption behaviors. In the SBO MP80 spent media, the peak area of penta-5AOS, hexa-5AOS, and abundant arabinose linkages was substantially smaller in the spent media than in the control, and the concentration of arabinose monomer was reduced (Fig. 5C; Table S5). Interestingly, 5AOS analysis revealed tetra-5AOS peak area is comparable with that of the control, suggesting that BPS MP80 does not import tetra-5AOS and has one or more extracellular endo-arabinofuranosidases that produce arabinose, arabinobiose, and/or arabinotriose from linear 5AOS. The peak area of 3,4-Xyl*p*/3,5-Ara*f* was smaller, and that of 3-Ara*f* slightly smaller, in the BPS MP80 spent media than in the control (Fig. 5C), implying that BPS MP80 tolerates single arabinose substitutions of 5AOS better than α-(1,3)-arabinofuranooligosaccharides.

In the spent media from BAL ATCC 15703, the peak area of all 4GOS, except for penta-4GOS, was smaller than in the control, and the peak area of 4-Gal and T-Gal linkages did not change. Possibly, most 4-Gal in SBO comes from non-linear 4GOS and/or galactosylated, non-galactan glycans. Additionally, the peak area of penta-5AOS, 5-Ara*f* (5-linked arabinose), and T-Ara*f* (terminal arabinose), and the concentration of arabinose were not different from the control (Fig. 5C; Table S5). It is therefore possible that the BAL ATCC 15703 grew on SBO primarily due to its utilization of a few GOS species.

### Arabinosidase content correlates with growth and consumption on SBO

Because arabinose and α-L-arabinofuranosyl linkages were common features of SBO, we hypothesized that the presence and number of α-arabinofuranosidase-containing glycoside hydrolase (GH) domains in a *Bifidobacterium* genome predict its ability to grow robustly on SBO as a sole carbon source. α-L-arabinofuranosidases are the most relevant GHs to robust SBO metabolism. *Bifidobacterium* α-L-arabinofuranosidases belong either to GH43, which comprises both α-arabinofuranosidases and β-xylopyranosidases, or to GH51, which comprises α-arabinofuranosidases specific for arabinoxylan and arabinoxylooligosaccharides (30).

**Fig 6:**
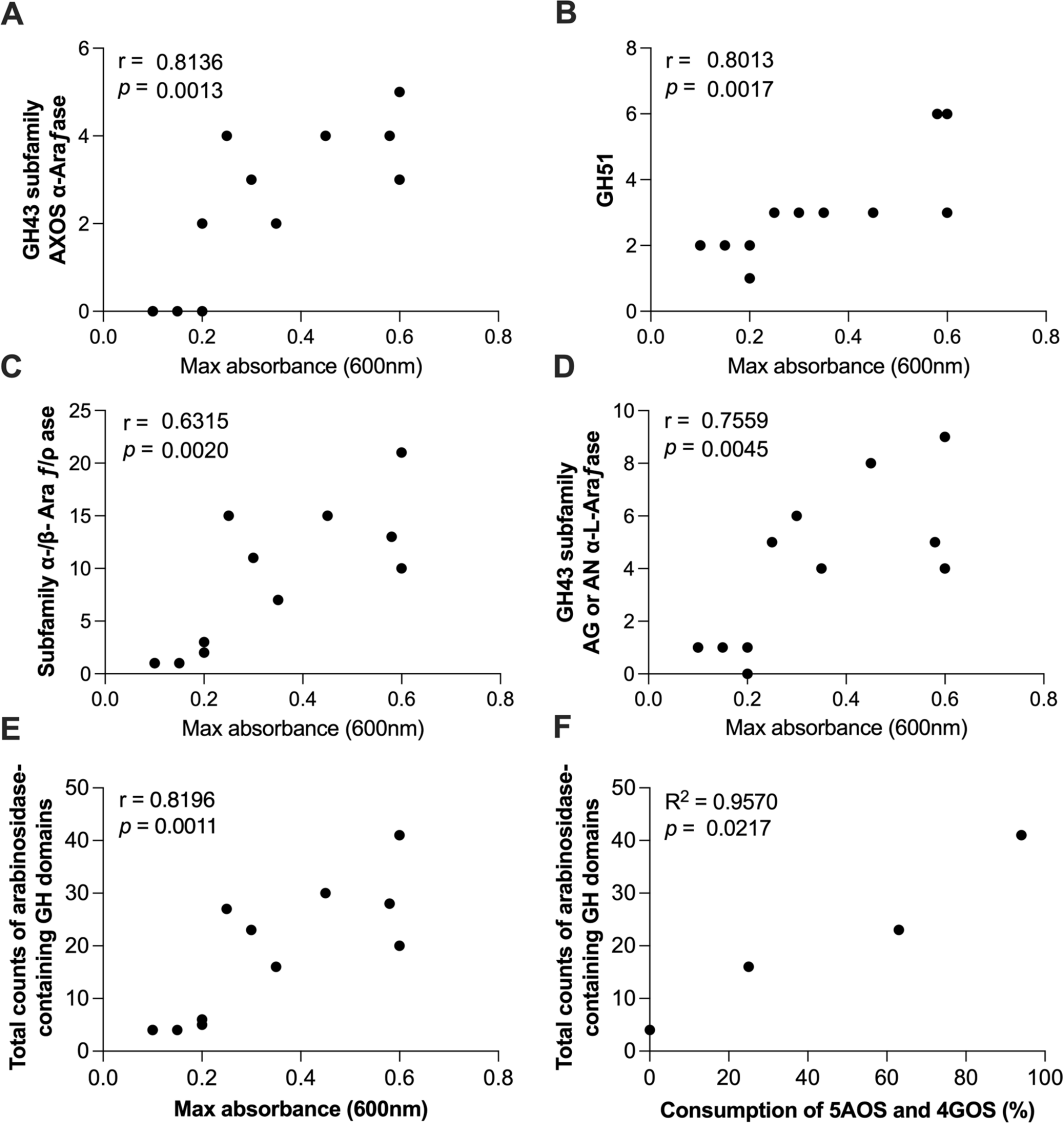
Fig 6. Arabinosidase content correlates with growth and consumption on SBO. ORFs from a total of 15 different *Bifidobacterium* strains were annotated, and GH43 and GH51 domains were tabulated. GH43 was grouped by a subfamily function. Strong correlations between maximum absorbance (600 nm) and (A) GH43 subfamily AXOS α-Araƒase, (B) GH51, (C) subfamily α-/β- Ara ƒ/ρase, and (D) GH43 subfamily AG or AN α-L-Araƒase were observed. All-domain count is strongly correlated with both the (E) maximum optical density in pure culture and (F) the consumption of 5AOS and 4GOS.

Open reading frames (ORFs) from a total of 15 different *Bifidobacterium* strains were annotated with dbCAN3 to find GHs, and GH43 and GH51 domains were tabulated in Table S6. GH43 was a subset by a subfamily function. Significant, strong, positive correlations were observed between counts of each GH domain and subfamily, and the maximum absorbance of their corresponding SBO culture (Fig. 6A to D). The strongest correlation observed was with the GH43 subfamily containing AXOS α-arabinofuranosidase (*r* = 0.8013, *P* = 0.0017; Fig. 6A). Furthermore, all-domain count is strongly correlated with both the maximum optical density in pure culture and the consumption of 5AOS and 4GOS (Fig. 6E and F).

### Two arabinofuranosidase clusters linked to catabolism of SBO and SBA

**Fig 7:**
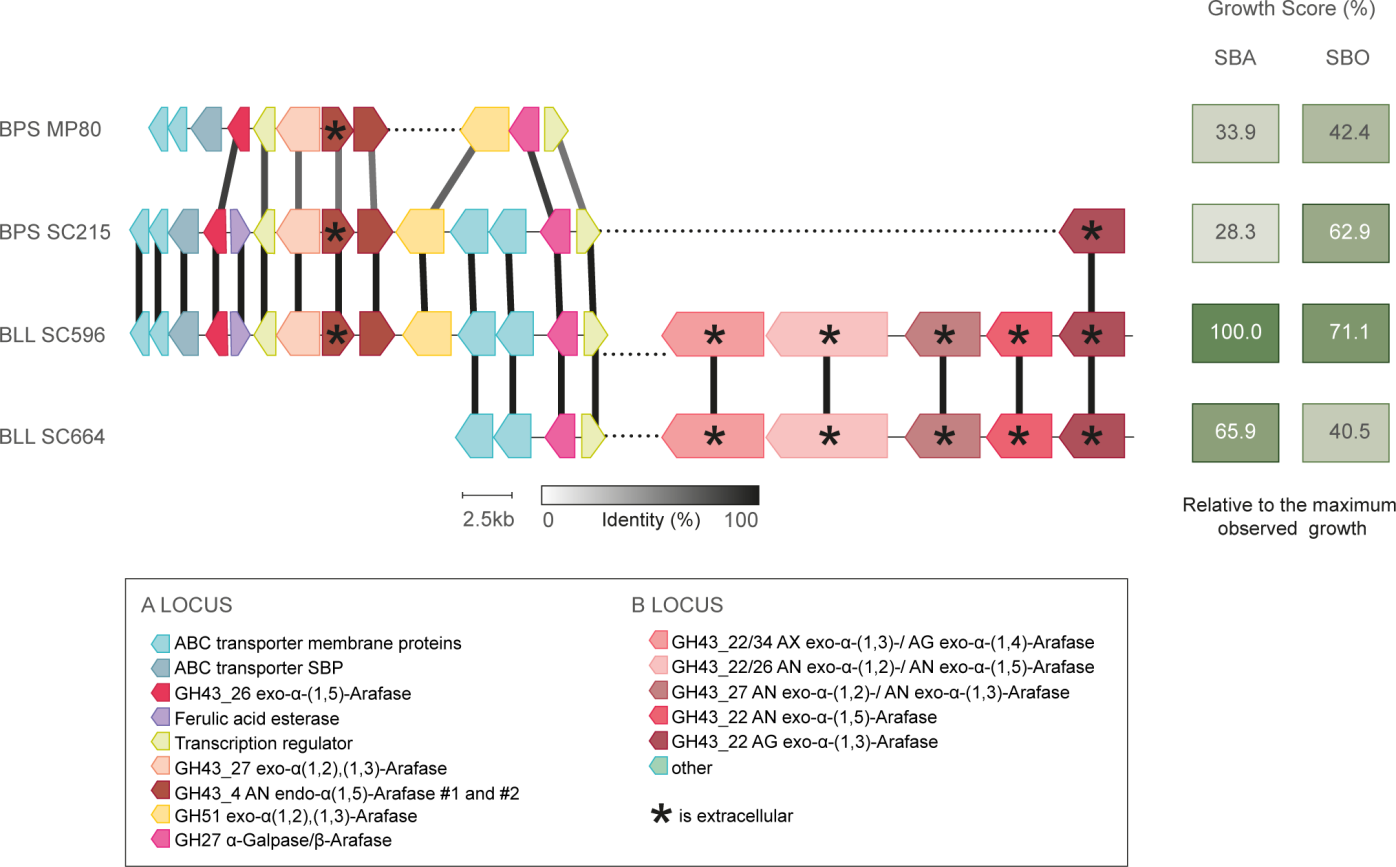
Synteny view of SBO-growing *Bifidobacterium* genomes containing an A locus (left side) and/or B locus (right side) arabinofuranosidases. Homologs are connected by a line shaded by percent amino acid identity (black being 100% amino acid identity). Growth % of each strain on SBA or SBO is presented relative to growth by BLL SC596 on SBA. The following characterized enzymes are tabulated in Table S5 and illustrated here: GH43_26 exo-α-(1,5)-Arafase (BLS DSM 20211 locus tag WP_007055573.1), GH43_27 exo-α-(1,2),(1,3)-Arafase (BLS DSM 20211 locus tag WP_007055569.1), both GH43_4 AN endo-α-(1,5)-Arafases (BLS DSM 20211 locus tags WP_032684165.1 and WP_032684164.1), GH51 exo-α-(1,2),(1,3)-Arafase (BLS DSM 20211 locus tag WP_080771011.1), GH43_22/34 AX exo-α-(1,3)-/ AG exo-α-(1,4)-Arafase (BLL JCM 1217 locus tag BLLJ_1850), GH43_22/26 AN exo-α-(1,2)-/ AN exo-α-(1,5)-Arafase (BLL JCM 1217 locus tag BLLJ_1851), GH43_27 AN exo-α-(1,2)-/ AN exo-α-(1,3)-Arafase (BLL JCM 1217 locus tag BLLJ_1852), GH43_22 AN exo-α-(1,5)-Arafase (BLL JCM 1217 locus tag BLLJ_1853), GH43_22 AG exo-α-(1,3)-Arafase (BLL JCM 1217 locus tag BLLJ_1854). “AX,” arabinoxylan; “AN,” arabinan; “AG,” arabinogalactan.

We previously demonstrated that growth on SBO by BLL SC596 and BPS MP80 resulted in an overt decrease in 5AOS, the main oligosaccharide type within the SBO pool. Yet, of these two strains, only BLL SC596 was able to grow on the polysaccharide SBA. Genetic analysis of BLL SC596 revealed two main clusters harboring arabinofuranosidases, one containing five extracellular GH43 enzymes (Fig. 7; B locus) and another containing three intracellular (GH51, GH43, GH27) and one extracellular (GH43) arabinofuranosidase (Fig. 7; A locus). Notably, the A locus also contains ABC transporter components putatively associated with transport of oligosaccharides. Genetic analysis of BPS MP80 indicates it solely possesses the A locus, suggesting that locus is responsible for the metabolism of 5AOS within the SBO pool. That BPS MP80 does not grow on polysaccharide SBA, while BLL SC596 does, suggests the B locus is primarily associated with extracellular arabinan degradation. This latter point also aligns with the lack of growth of BLL SC215 on SBA but shows good growth on SBO (Fig. 4).

### SBO enables BLL SC596 to selectively persist in fecal fermentations

**Fig 8:**
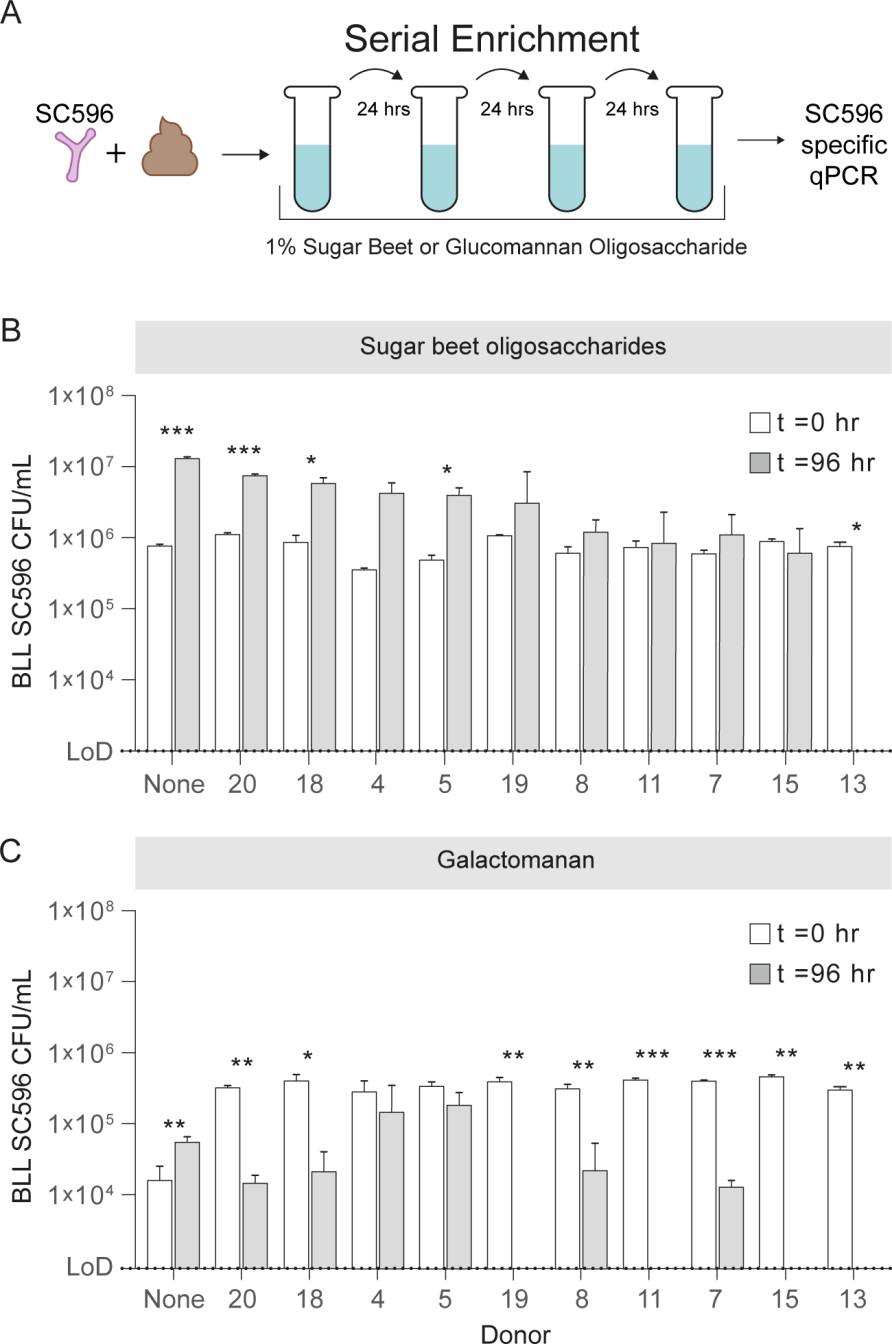
(**A**) Diagram of the serial fecal fermentations to test the persistence of BLL SC596 in combination with (**B**) SBO or (**C**) galactomannan as sole carbon source using 10 different donor feces. Bacterial counts (CFU/mL) of BLL SC596 (interpolated by qPCR) are shown immediately after inoculation (gray bar) and after three serial passages (white bar) for each serial fermentation. Error bars represent the average of three fermentations. LoD, limit of detection; NS, not significant; ***P < 0.001; ** 0.001 < *P* < 0.01; * 0.01 < *P* < 0.05.

Given the robust growth of BLL SC596 on SBO, we next examined if synbiotic application of the two components facilitated the persistence of BLL SC596 in fecal fermentations. A serial fecal fermentation assay was performed as previously described (Fig. 8A; [31]), wherein BLL SC596 was combined with SBO or galactomannan, the latter of which does not support the growth of BLL SC596 (data not shown). Triplicate fermentations inoculated with 10 different donor feces were carried out, and strain-specific qPCR was performed after the third consecutive fermentation. When supplemented with SBO, BLL SC596 successfully persisted after three fermentation cycles in 9 of 10 donors (Fig. 8B). Of those fermentations where BLL SC596 persisted, four resulted in higher levels of BLL SC596 than the initial concentration while three resulted in equivalent levels to the starting BLL SC596 levels. Conversely, BLL SC596 did not persist in 8 of 10 fermentations where galactomannan was used as the synbiotic partner glycan (Fig. 8C). Because galactomannan does not support BLL SC596 for growth, some yet-to-be determined mechanism, such as cross-feeding of galactomannan components, is likely responsible for the persistence of BLL SC596 in 2 of the 10 fermentations.

## DISCUSSION

Characterizing the mechanisms by which human gut bacteria degrade dietary fiber for their selective metabolism is an inherently complex task. Polysaccharide degradation in the gut occurs via diverse networks of microorganisms driven by primary degraders that directly catabolize larger glycans for growth but also create “released” substrates, such as mono-, di-, and oligosaccharides, which, in turn, cross-feed secondary consumers in a process termed “substrate cross-feeding” (32). Recent advances in high-throughput glycomics have revealed that food fibers are quite diverse, possessing a range of monosaccharide and linkage compositions creating a challenge to explore substrate cross-feeding interactions from a larger range of fiber substrates. Recently, Paviani and coworkers developed a novel method, termed Fenton’s Initiation Toward Defined Oligosaccharide Groups (FITDOG; [6]), to create oligosaccharide pools from any polysaccharide substrate, a method that was later shown to be scalable (10). In this pilot study, we examined the product of the FITDOG reaction on sugar beet pulp. Compositionally, the FITDOG oligosaccharide products of SBP were representative of their polysaccharide parents with oligosaccharides representing the majority of arabinan fiber. Thus, the FITDOG-derived SBOs used here represent not only unique prebiotic oligosaccharide pools but also novel probes to interrogate fiber–bacterial interactions.

Fecal fermentations of SBP and SBO using 10 different donors reinforced the concept that individual gut community structure drives inter-individual variation in how a gut community responds to a carbohydrate structure. However, several trends were readily observed. First, *Bifidobacterium* and *Bacteroides* populations, genera known to be enriched by sugar beet arabinan (28), were enriched by both SBP and SBO. Secondly, SBO fermentations generally exhibited a more rapid drop in pH than the SBP fermentations suggesting that the oligosaccharide substrate was more readily consumed by the fecal community. This concept is supported by the fact that more individual bifidobacterial strains could grow on SBO as a substrate than sugar beet arabinan, the latter being the dominant polysaccharide type in SBP. Numerous researchers have demonstrated that creation of mono- and oligosaccharides by extracellular degradation from a primary consumer can cross-feed bifidobacteria (33). This includes extracellular cleavage by *Bacteroides* of plant glycans such as inulin (34), β-mannans (35), galactomannans (36), and arabinogalactans (37) into smaller components, which, in turn, cross-fed select bifidobacterial strains. Such cross-feeding can also occur between bifidobacterial strains such as extracellular cleavage of mucin by *B. bifidum*, resulting in the provision of smaller components (oligo-, di-, and monosaccharides) to non-mucin-consuming strains (38). It remains to be determined if the strain diversity within SBO-enriched communities is indeed more diverse than that derived from SBP-enriched communities.

Glycomic analysis of individual strains grown on SBO revealed that different components of the same SBO pool were used by individual bifidobacterial strains. For example, BLL SC596 consumed the majority of the SBO pool while BPS MP80 consumed the arabinooligosaccharide SBO component and BAL ATCC 15703 consumed the GOS SBO component. A similarly specific GOS consumption was recently observed where *Bacteroides cellulosilyticus* grown on arabinogalactan cross-fed GOS to *B. breve* UCC2003 (39). These examples illustrate the complexity in defining secondary consumer networks in fiber degradation given a single polysaccharide can produce multiple downstream oligosaccharide components that differentially feed bacterial subpopulations.

Genomic analysis of the bifidobacterial strains that grew on arabinan and SBO enabled insight into specific arabinan consumption patterns. Others have shown that arabinan degradation requires a synergistic combination of endo-arabinofuranosidaseas and exo-arabinases (40). The extracellular arabinofuranosidase cluster identified in this work (B locus; Fig. 7) is homologous to the previously characterized arabinan-debranching enzymes encoded by other BLL strains (41–43). Growth on arabinan coincided with the presence of the B locus, a correlation also identified previously (44). Recently, Zhang and co-workers showed that deletions within this cluster prevented growth on arabinan both *in vitro* and *in vivo* (45). However, growth on SBO correlated with either the A or B locus; however, a strain possessing both loci exhibited the most growth. The A locus identified here contains mostly intracellular enzymes and is homologous to the previously characterized arabinan-degrading enzymes encoded by *B. longum* subsp. *suis* ATCC 27533 (40). Glycomics data suggest subtle differences in SBO consumption behavior linked to these two loci; for example, BLL SC596 (A+, B+) was able to consume α-(1,5)-arabinofuranotetraose while BPS MP80 (A+, B-) was not. Thus, differential combinations of genetic content, enzyme specificity, and expression behaviors likely drive these different phenotypes.

The ability of FITDOG to generate oligosaccharides from any fiber provides a unique opportunity to decipher specific fiber degradation processes by individual microbes or microbial guilds across a much wider range of fiber compositions (monosaccharide, linkage, etc.) inherent to human and animal diets. Furthermore, several groups have demonstrated that co-supplementation of a fiber combined with a fiber-consuming microbe enables persistent colonization in hosts (45, 46). Others have shown that supplementation of oligosaccharides combined with an oligosaccharide-consuming bifidobacteria enables persistence, and in some cases dominance, of the strain in hosts (46–49). Kok and coworkers (31) recently demonstrated *in vitro* persistence of a select BLL strain when combined with XOS supplementation through serial passage of fecal enrichments. This work demonstrates a similar outcome wherein a strain identified to grow well on SBO (BLL SC596) was shown to persist in serial fecal fermentations supplemented with SBO. Given the range of possible oligosaccharides that can be obtained from diverse fibers with different monosaccharide and linkage content, this approach provides a unique landscape to explore synergistic interactions between naturally derived fibers and native microbial strains that are well adapted to the competitive gut environment, revealing excellent candidates for synergistic synbiotic pairs.

## Data Availability

The raw 16S sequencing data have been deposited in the NCBI Sequence Read Archive (SRA) under the BioProject accession number PRJNA816918.

## SUPPLEMENTAL MATERIAL

Supplemental material aem.02077-24-s0001.pdf Tables S1 to S6 and Figures S1 to S3.

ASM does not own the copyrights to Supplemental Material that may be linked to, or accessed through, an article. The authors have granted ASM a non-exclusive, world-wide license to publish the Supplemental Material files. Please contact the corresponding author directly for reuse.

## References

[1] Krumbeck JA, Maldonado-Gomez MX, Ramer-Tait AE, Hutkins RW. 2016. Prebiotics and synbiotics: dietary strategies for improving gut health. Curr Opin Gastroenterol 32:110–119. doi:10.1097/MOG.0000000000000249 26825589

[2] Delannoy-Bruno O, Desai C, Castillo JJ, Couture G, Barve RA, Lombard V, Henrissat B, Cheng J, Han N, Hayashi DK, Meynier A, Vinoy S, Lebrilla CB, Marion S, Heath AC, Barratt MJ, Gordon JI. 2022. An approach for evaluating the effects of dietary fiber polysaccharides on the human gut microbiome and plasma proteome. Proc Natl Acad Sci U S A 119:e2123411119. doi:10.1073/pnas.2123411119 35533274 PMC9171781

[3] Deehan EC, Walter J. 2016. The fiber gap and the disappearing gut microbiome: implications for human nutrition. Trends Endocrinol Metab 27:239–242. doi:10.1016/j.tem.2016.03.001 27079516

[4] Rooks MG, Garrett WS. 2016. Gut microbiota, metabolites and host immunity. Nat Rev Immunol 16:341–352. doi:10.1038/nri.2016.42 27231050 PMC5541232

[5] Sonnenburg ED, Smits SA, Tikhonov M, Higginbottom SK, Wingreen NS, Sonnenburg JL. 2016. Diet-induced extinctions in the gut microbiota compound over generations. Nature 529:212–215. doi:10.1038/nature16504 26762459 PMC4850918

[6] Amicucci MJ, Nandita E, Galermo AG, Castillo JJ, Chen S, Park D, Smilowitz JT, German JB, Mills DA, Lebrilla CB. 2020. A nonenzymatic method for cleaving polysaccharides to yield oligosaccharides for structural analysis. Nat Commun 11:3963. doi:10.1038/s41467-020-17778-1 32770134 PMC7414865

[7] Amicucci MJ, Galermo AG, Nandita E, Vo T-T, Liu Y, Lee M, Xu G, Lebrilla CB. 2019. A rapid-throughput adaptable method for determining the monosaccharide composition of polysaccharides. Int J Mass Spectrom 438:22–28. doi:10.1016/j.ijms.2018.12.009

[8] Galermo AG, Nandita E, Castillo JJ, Amicucci MJ, Lebrilla CB. 2019. Development of an extensive linkage library for characterization of carbohydrates. Anal Chem 91:13022–13031. doi:10.1021/acs.analchem.9b03101 31525948 PMC9759349

[9] Couture G, Cheang SE, Suarez C, Chen Y, Bacalzo NP, Jiang J, Weng C-Y, Stacy A, Castillo JJ, Delannoy-Bruno O, Webber DM, Barratt MJ, Gordon JI, Mills DA, German JB, Fukagawa NK, Lebrilla CB. 2024. A multi-glycomic platform for the analysis of food carbohydrates. Nat Protoc 19:3321–3359. doi:10.1038/s41596-024-01017-8 39026121

[10] Paviani B, Masarweh C, Bhattacharya M, Ozturk G, Castillo J, Couture G, Lebrilla CB, Mills DA, Barile D. 2024. Eat your beets: conversion of polysaccharides into oligosaccharides for enhanced bioactivity. Int J Biol Macromol 256:128472. doi:10.1016/j.ijbiomac.2023.128472 38029906

[11] Gibson GR, Hutkins R, Sanders ME, Prescott SL, Reimer RA, Salminen SJ, Scott K, Stanton C, Swanson KS, Cani PD, Verbeke K, Reid G. 2017. Expert consensus document: The International Scientific Association for Probiotics and Prebiotics (ISAPP) consensus statement on the definition and scope of prebiotics. Nat Rev Gastroenterol Hepatol 14:491–502. doi:10.1038/nrgastro.2017.75 28611480

[12] Castillo JJ, Couture G, Bacalzo NP, Chen Y, Chin EL, Blecksmith SE, Bouzid YY, Vainberg Y, Masarweh C, Zhou Q, Smilowitz JT, German JB, Mills DA, Lemay DG, Lebrilla CB. 2022. The development of the Davis food glycopedia-A glycan encyclopedia of food. Nutrients 14:1639. doi:10.3390/nu14081639 35458202 PMC9032246

[13] de Moura Bell JMLN, Cohen JL, de Aquino LFMC, Lee H, de Melo Silva VL, Liu Y, Domizio P, Barile D. 2018. An integrated bioprocess to recover bovine milk oligosaccharides from colostrum whey permeate. J Food Eng 216:27–35. doi:10.1016/j.jfoodeng.2017.07.022 29217872 PMC5714328

[14] Sinrod AJG, Li X, Bhattacharya M, Paviani B, Wang SC, Barile D. 2021. A second life for wine grapes: discovering potentially bioactive oligosaccharides and phenolics in chardonnay marc and its processing fractions. LWT 144:111192. doi:10.1016/j.lwt.2021.111192

[15] Nandita E, Bacalzo NP, Ranque CL, Amicucci MJ, Galermo A, Lebrilla CB. 2021. Polysaccharide identification through oligosaccharide fingerprinting. Carbohydr Polym 257:117570. doi:10.1016/j.carbpol.2020.117570 33541630 PMC9674106

[16] De MAN JC, Rogosa M, Sharpe ME. 1960. A medium for the cultivation of lactobacilli. J Appl Bacteriol 23:130–135. doi:10.1111/j.1365-2672.1960.tb00188.x

[17] Pérez-Burillo S, Molino S, Navajas-Porras B, Valverde-Moya ÁJ, Hinojosa-Nogueira D, López-Maldonado A, Pastoriza S, Rufián-Henares JÁ. 2021. An in vitro batch fermentation protocol for studying the contribution of food to gut microbiota composition and functionality. Nat Protoc 16:3186–3209. doi:10.1038/s41596-021-00537-x 34089022

[18] Zheng J, Ge Q, Yan Y, Zhang X, Huang L, Yin Y. 2023. dbCAN3: automated carbohydrate-active enzyme and substrate annotation. Nucleic Acids Res 51:W115–W121. doi:10.1093/nar/gkad328 37125649 PMC10320055

[19] Gilchrist CLM, Chooi Y-H. 2021. Clinker & clustermap.js: automatic generation of gene cluster comparison figures. Bioinformatics 37:2473–2475. doi:10.1093/bioinformatics/btab007 33459763

[20] Walker AW, Duncan SH, McWilliam Leitch EC, Child MW, Flint HJ. 2005. pH and peptide supply can radically alter bacterial populations and short-chain fatty acid ratios within microbial communities from the human colon. Appl Environ Microbiol 71:3692–3700. doi:10.1128/AEM.71.7.3692-3700.2005 16000778 PMC1169066

[21] Davis JCC, Lewis ZT, Krishnan S, Bernstein RM, Moore SE, Prentice AM, Mills DA, Lebrilla CB, Zivkovic AM. 2017. Growth and morbidity of gambian infants are influenced by maternal milk oligosaccharides and infant gut microbiota. Sci Rep 7:40466. doi:10.1038/srep40466 28079170 PMC5227965

[22] Bolyen E, Rideout JR, Dillon MR, Bokulich NA, Abnet CC, Al-Ghalith GA, Alexander H, Alm EJ, Arumugam M, Asnicar F, et al.. 2019. Reproducible, interactive, scalable and extensible microbiome data science using QIIME 2. Nat Biotechnol 37:852–857. doi:10.1038/s41587-019-0209-9 31341288 PMC7015180

[23] Oksanen J, Kindt R, Legendre P, O’Hara B, Stevens MHH, Oksanen MJ, Suggests M. 2007. The vegan package.Community ecology package

[24] Wickham H. 2016. Ggplot2 - Elegant graphics for data analysis. Springer-Verlag, New York, New York, NY.

[25] Koressaar T, Remm M. 2007. Enhancements and modifications of primer design program Primer3. Bioinformatics 23:1289–1291. doi:10.1093/bioinformatics/btm091 17379693

[26] Weng C-Y, Suarez C, Cheang SE, Couture G, Goodson ML, Barboza M, Kalanetra KM, Masarweh CF, Mills DA, Raybould HE, Lebrilla CB. 2024. Quantifying gut microbial short-chain fatty acids and their isotopomers in mechanistic studies using a rapid, readily expandable LC–MS platform. Anal Chem 96:2415–2424. doi:10.1021/acs.analchem.3c04352 38288711 PMC10867797

[27] Brodkorb A, Egger L, Alminger M, Alvito P, Assunção R, Ballance S, Bohn T, Bourlieu-Lacanal C, Boutrou R, Carrière F, et al.. 2019. INFOGEST static in vitro simulation of gastrointestinal food digestion. Nat Protoc 14:991–1014. doi:10.1038/s41596-018-0119-1 30886367

[28] Al-Tamimi M, Palframan RJ, Cooper JM, Gibson GR, Rastall RA. 2006. In vitro fermentation of sugar beet arabinan and arabino-oligosaccharides by the human gut microflora. J Appl Microbiol 100:407–414. doi:10.1111/j.1365-2672.2005.02780.x 16430518

[29] Mattarelli P, Biavati B. 2018. Species in the genus bifidobacterium, p 9–48. In The bifidobacteria and related organisms. Elsevier.

[30] Lagaert S, Pollet A, Delcour JA, Lavigne R, Courtin CM, Volckaert G. 2010. Substrate specificity of three recombinant α-L-arabinofuranosidases from bifidobacterium adolescentis and their divergent action on arabinoxylan and arabinoxylan oligosaccharides. Biochem Biophys Res Commun 402:644–650. doi:10.1016/j.bbrc.2010.10.075 20971079

[31] Kok CR, Gomez Quintero DF, Niyirora C, Rose D, Li A, Hutkins R. 2019. An in vitro enrichment strategy for formulating synergistic synbiotics. Appl Environ Microbiol 85:e01073-19. doi:10.1128/AEM.01073-19 31201276 PMC6677857

[32] Smith NW, Shorten PR, Altermann E, Roy NC, McNabb WC. 2019. The classification and evolution of bacterial cross-feeding. Front Ecol Evol 7. doi:10.3389/fevo.2019.00153

[33] Xiao M, Zhang C, Duan H, Narbad A, Zhao J, Chen W, Zhai Q, Yu L, Tian F. 2024. Cross-feeding of bifidobacteria promotes intestinal homeostasis: a lifelong perspective on the host health. NPJ Biofilms Microbiomes 10:47. doi:10.1038/s41522-024-00524-6 38898089 PMC11186840

[34] Falony G, Calmeyn T, Leroy F, De Vuyst L. 2009. Coculture fermentations of Bifidobacterium species and Bacteroides thetaiotaomicron reveal a mechanistic insight into the prebiotic effect of inulin-type fructans. Appl Environ Microbiol 75:2312–2319. doi:10.1128/AEM.02649-08 19251883 PMC2675216

[35] Gao G, Cao J, Mi L, Feng D, Deng Q, Sun X, Zhang H, Wang Q, Wang J. 2021. BdPUL12 depolymerizes β-mannan-like glycans into mannooligosaccharides and mannose, which serve as carbon sources for Bacteroides dorei and gut probiotics. Int J Biol Macromol 187:664–674. doi:10.1016/j.ijbiomac.2021.07.172 34339781

[36] Mary PR, Kapoor M. 2022. Co-culture fermentations suggest cross-feeding among Bacteroides ovatus DSMZ 1896, Lactiplantibacillus plantarum WCFS1 and Bifidobacterium adolescentis DSMZ 20083 for utilizing dietary galactomannans. Food Res Int 162:111942. doi:10.1016/j.foodres.2022.111942 36461198

[37] Wang Y, LaPointe G. 2020. Arabinogalactan utilization by Bifidobacterium longum subsp. longum NCC 2705 and Bacteroides caccae ATCC 43185 in monoculture and coculture. Microorganisms 8:1703. doi:10.3390/microorganisms8111703 33142707 PMC7693162

[38] Turroni F, Milani C, Duranti S, Mahony J, van Sinderen D, Ventura M. 2018. Glycan utilization and cross-feeding activities by Bifidobacteria. Trends Microbiol 26:339–350. doi:10.1016/j.tim.2017.10.001 29089173

[39] Munoz J, James K, Bottacini F, Van Sinderen D. 2020. Biochemical analysis of cross-feeding behaviour between two common gut commensals when cultivated on plant-derived arabinogalactan. Microb Biotechnol 13:1733–1747. doi:10.1111/1751-7915.13577 32385941 PMC7533333

[40] Kang Y, Choi C-Y, Kang J, Ju Y-R, Kim HB, Han NS, Kim T-J. 2024. Functional characterization of endo- and exo-hydrolase genes in arabinan degradation gene cluster of Bifidobacterium longum subsp. suis. IJMS 25:3175. doi:10.3390/ijms25063175 38542148 PMC10970622

[41] Sasaki Y, Matsuo A, Hashiguchi M, Fujimura K, Koshino H, Tanaka K, Ito Y, Kitahara K, Ishiwata A, Fujita K. 2025. Structural analysis of gum arabic side chains from acacia seyal released by bifidobacterial β-arabino-oligosaccharide 3-O-β-L-arabinopyranosyl-α-L-arabinofuranosidase. Carbohydr Polym 349:122965. doi:10.1016/j.carbpol.2024.122965 39643419

[42] Komeno M, Hayamizu H, Fujita K, Ashida H. 2019. Two novel α-l-arabinofuranosidases from Bifidobacterium longum subsp. longum belonging to glycoside hydrolase family 43 Cooperatively Degrade Arabinan. Appl Environ Microbiol 85:e02582-18. doi:10.1128/AEM.02582-18 30635377 PMC6414367

[43] Komeno M, Yoshihara Y, Kawasaki J, Nabeshima W, Maeda K, Sasaki Y, Fujita K, Ashida H. 2022. Two α-L-arabinofuranosidases from Bifidobacterium longum subsp. longum are involved in arabinoxylan utilization. Appl Microbiol Biotechnol 106:1957–1965. doi:10.1007/s00253-022-11845-x 35235007

[44] Arboleya S, Bottacini F, O’Connell-Motherway M, Ryan CA, Ross RP, van Sinderen D, Stanton C. 2018. Gene-trait matching across the Bifidobacterium longum pan-genome reveals considerable diversity in carbohydrate catabolism among human infant strains. BMC Genomics 19:33. doi:10.1186/s12864-017-4388-9 29310579 PMC5759876

[45] Zhang C, Yu L, Ma C, Jiang S, Zhang Y, Wang S, Tian F, Xue Y, Zhao J, Zhang H, Liu L, Chen W, Huang S, Zhang J, Zhai Q. 2023. A key genetic factor governing arabinan utilization in the gut microbiome alleviates constipation. Cell Host Microbe 31:1989–2006. doi:10.1016/j.chom.2023.10.011 37992712

[46] Shepherd ES, DeLoache WC, Pruss KM, Whitaker WR, Sonnenburg JL. 2018. An exclusive metabolic niche enables strain engraftment in the gut microbiota. Nature 557:434–438. doi:10.1038/s41586-018-0092-4 29743671 PMC6126907

[47] Button JE, Autran CA, Reens AL, Cosetta CM, Smriga S, Ericson M, Pierce JV, Cook DN, Lee ML, Sun AK, Alousi AM, Koh AY, Rechtman DJ, Jenq RR, McKenzie GJ. 2022. Dosing a synbiotic of human milk oligosaccharides and B. infantis leads to reversible engraftment in healthy adult microbiomes without antibiotics. Cell Host Microbe 30:712–725. doi:10.1016/j.chom.2022.04.001 35504279

[48] Heiss BE, Ehrlich AM, Maldonado-Gomez MX, Taft DH, Larke JA, Goodson ML, Slupsky CM, Tancredi DJ, Raybould HE, Mills DA. 2021. Bifidobacterium catabolism of human milk oligosaccharides overrides endogenous competitive exclusion driving colonization and protection. Gut Microbes 13:1986666. doi:10.1080/19490976.2021.1986666 34705611 PMC8555557

[49] Krumbeck JA, Maldonado-Gomez MX, Martínez I, Frese SA, Burkey TE, Rasineni K, Ramer-Tait AE, Harris EN, Hutkins RW, Walter J. 2015. In vivo selection to identify bacterial strains with enhanced ecological performance in synbiotic applications. Appl Environ Microbiol 81:2455–2465. doi:10.1128/AEM.03903-14 25616794 PMC4357922

