# Supplementary material for: Generation of novel prebiotic oligosaccharide pools from fiber drives biological insight in bacterial glycan metabolism
Source: Appl Environ Microbiol. 2025 Feb 6;91(3):e02077-24. doi: 10.1128/aem.02077-24 (PMC11921329; doi:10.1128/aem.02077-24)

Supplementary Table 1. Within-donor permutational ANOVA of Bray-Curtis community dissimilarities between inocula and 24 and 48 hour samples.

	Adonis			Beta-dispersion
Donor	R^2	pseudo-F	*p*-value	*p*-value
8	0.96	353.89	0.00025 ***	0.162959
10	0.73	45.17	0.00025 ***	0.00075 ***
4	0.65	32.17	0.00025 ***	0.00025 ***
7	0.63	28.91	0.00025 ***	0.00075 ***
13	0.62	28.01	0.00025 ***	0.00025 ***
5	0.60	25.70	0.00025 ***	0.00025 ***
11	0.57	22.76	0.00025 ***	0.00025 ***
19	0.56	20.40	5e-04 ***	0.00025 ***
3	0.52	18.57	0.00025 ***	0.00025 ***
2	0.50	16.06	0.00025 ***	0.00025 ***

*** p < 0.001

Supplementary Table 2. Supplementary Table 2: per-donor comparison of 24/48 hrs SBO and SBP fermentations using PERMANOVA of Bray-Curtis dissimilarities

	Adonis			Beta-dispersion
Donor	R^2	pseudo-F	*p*-value	*p*-value
4	0.84	51.57	0.0015 **	0.4736
19	0.82	39.97	0.0015 **	0.00025 ***
7	0.79	36.90	0.002 **	0.0445 *
11	0.77	32.75	0.003 **	0.0810
10	0.73	27.54	0.002 **	0.0645
2	0.57	12.01	0.003 **	0.7298
3	0.37	5.90	0.00175 **	0.0047 **
8	0.29	3.75	0.00775 **	0.1245
13	0.28	3.81	0.01874 *	0.6863
5	0.21	2.60	0.0660	0.7213

* 0.01 < p < 0.05; ** 0.001 < p < 0.01; *** p < 0.001

Supplementary Table 3. Statistical comparison by t-test of the difference of the mean total SCFA concentration and mean pH, respectively, of each donor's SBO and SBP fermentations, respectively.

Donor	2	3	4	5	7	8	10	11	13	19
Total SCFA										
SBO mean μg/mL	11,477	9,803	6,694	10,230	11,294	3,293	16,611	13,810	5,880	7,783
SBP mean μg/mL	11,741	12,546	4,863	9,082	9,223	4,998	7,279	7,178	8,583	7,500
t-test *p*-value	0.78	0.10	0.06	0.76	0.11	0.046*	0.0036**	0.0014**	0.017*	0.65
Final pH										
SBO mean pH	5.96	5.94	5.95	5.85	5.99	6.28	6.08	6.05	6.13	5.82
SBP mean pH	6.00	5.90	6.05	5.95	6.14	5.95	6.06	6.30	5.97	5.86
t-test *p*-value	0.07	0.68	0.14	0.45	0.027*	0.040*	0.89	0.08	0.031*	0.71

* $p < 0.05$; ** $p < 0.01$

Supplementary Table 4. Growth scores for each *Bifidobacterium* strains calculated based on the maximum optical density observed in pure cultures supplemented with SBA or SBO. The growth scores are calculated by dividing the strain's max OD by the best fermenter's max OD and multiplying by 100. Consequently, growth scores, expressed in percentage, represent how well the strain grew relative to the maximum growth recorded (SC596). Prior to collecting data, discrete growth characterizations (good, moderate, and no growth) were assigned to arbitrary ranges of growth scores as follows: no growth 0-20, poor 21-35, moderate 36-60, and good 61-100%.

**Genera and Specie**	**Specie acronym**	**Strain**	**Glycan**	**Avg Max OD**	**Growth Score**	
					**(%)**	**Term**
B. longum subsp. longum	BLL	SC596	SBA	0.8685	100.00	good
B. longum subsp. longum	BLL	SC596	SBO	0.6175	71.10	good
B. longum subsp. longum	BLL	15439	SBO	0.5970	68.74	good
B. longum subsp. longum	BLL	SC664	SBA	0.5720	65.86	good
B. pseudocatenulatum	BPS	SC585	SBO	0.5635	64.88	good
B. adolescentis	BAD	SC215	SBO	0.5460	62.87	good
B. animalis subsp. l*actis*	BAL	27536	SBO	0.4165	47.96	moderate
B. pseudocatenulatum	BPS	15703	SBO	0.3860	44.44	moderate
B. longum subsp. longum	BLL	MP80	SBO	0.3685	42.43	moderate
B. catenulatum subsp. kashiwanohense	BCK	SC664	SBO	0.3520	40.53	moderate
B. breve	BBR	SC95	SBO	0.3085	35.52	moderate
B. adolescentis	BAD	15703	SBA	0.3000	34.54	poor
B. pseudocatenulatum	BPS	MP80	SBA	0.2945	33.91	poor
B. longum subsp. *infantis*	BLI	15697	SBO	0.2685	30.92	poor
B. longum subsp. longum	BLL	SC215	SBA	0.2455	28.27	poor
B. catenulatum subsp. kashiwanohense	BCK	15439	SBA	0.2080	23.95	poor
B. bifidum	BBI	SC555	SBO	0.1320	15.20	no growth
B. bifidum	BBI	SC555	SBA	0.1315	15.14	no growth
B. bifidum	BBI	29521	SBO	0.126	14.51	no growth
Negative control	NA	no inoc	SBO	0.0965	11.11	no growth
Negative control	NA	no inoc	basal	0.0955	11.00	no growth
Negative control	NA	no-inoc	SBA	0.0950	10.94	no growth
Negative control	NA	no-inoc	SBO	0.0870	10.02	no growth

Supplementary Table 5. Statistical comparison of (A) the concentration of total monosaccharides reported in mg/ml (B) peak area of product ions representing α-(1,5)-AOS and β-(1,4)-GOS, and (C) measured glycosidic linkages in the spent media of pure cultures supplemented with SBO. Each strain was grown in triplicate. Differences were evaluated with ANOVA and Dunnett multiple comparisons were BBI SC555 was used as the reference group.

A) Total monosaccharides (mg/ml)

	Ara	Xyl	Gal	GalA	Glc	Fru	Rha	GlcA	Man	Rib	other
Mean											
BBI SC555	1.745	0.081	0.735	0.488	0.364	0.021	0.150	0.015	0.190	0.096	0.063
BAD 15703	2.002	0.090	0.969	0.511	0.435	0.079	0.141	0.048	0.192	0.114	0.057
BPS MP80	1.237	0.107	0.980	0.626	0.428	0.072	0.226	0.072	0.230	0.107	0.057
BLL SC596	0.301	0.298	0.691	0.580	0.429	0.395	0.146	0.105	0.140	0.092	0.054
Adjusted p-value. Compared to BBI											
BAD 15703	0.445	0.944	0.173	0.979	0.304	0.081	0.925	0.628	>0.9999	0.526	0.661
BPS MP80	0.004**	0.651	0.188	0.357	0.280	0.102	0.196	0.099	0.693	0.952	0.641
BLL SC596	0.003**	0.729	0.946	0.871	0.986	0.563	1.000	0.650	0.472	0.993	0.504

B) Product ions representing α-(1,5)-arabinofuranooligosaccharides and β-(1,4)-galactopyranooligosaccharides (Peak Areas)

	tetra5A	penta-5A	hexa-5A	hepta-5A	octa-5A	tetra-4G	penta-4G	hexa-4G	hepta-4G
Mean									
BBI SC555	1360970	1634012	589607	159062	0	198334	127755	59108	32411
BAD 15703	1084267	1470098	538604	22345	0	14194	36558	7245	5660
BPS MP80	948728	172507	34701	20072	0	12894	115432	60188	23627
BLL SC596	61695	34189	20797	4468	0	4669	11989	1540	465.7
Adjusted p-value. Compared to BBI									
BAD 15703	0.246	0.540	0.903	0.308	NA	0.010**	0.005**	0.013*	0.082
BPS MP80	0.108	0.014*	0.008**	0.304	NA	0.009**	0.526	0.999	0.461
BLL SC596	0.016*	0.011*	0.007**	0.256	NA	0.008**	0.011*	0.011*	0.056

C) Glycosidic linkages (Peak Areas)

	T-Araf	5-Araf	3-Araf	2-Araf	T-Arap	2,3-Araf	3,4Xylp or 3,5-Araf	4-Gal	T-Gal	4-Glc	T-Glc
Mean											
BBI SC555	107235	18900	4644	2560	1208	706.3	5835	3445	6863	2872	4868
BAD 15703	123968	22723	5006	2510	1378	757.3	6988	3680	6259	3128	4306
BPS MP80	38112	4889	2365	2241	2346	173.3	1049	5405	11230	4386	7327
BLL SC596	23623	1942	2130	1838	1567	66.33	611.3	1863	8953	4358	5251
Adjusted p-value. Compared to BBI											
BAD 15703	0.588	0.581	0.791	0.996	0.698	0.986	0.585	0.921	0.964	0.909	0.615
BPS MP80	0.005**	<0.0001****	0.032*	0.772	0.052	0.131	0.0003***	0.220	0.305	0.374	0.365
BLL SC596	0.001***	<0.0001****	0.001**	0.039*	0.290	0.091	0.005**	0.001***	0.077	0.197	0.904

	T-Xylp	T-Rha	T-Man	X-Hex	T-Fuc	other
Mean						
BBI SC555	1386	1303	2141	2074	992.7	2135
BAD 15703	1782	1778	2265	2976	1766	2958
BPS MP80	2404	1589	4161	2736	1929	4113
BLL SC596	1937	2452	3091	1015	2252	2402
Adjusted p-value. Compared to BBI						
BAD 15703	0.441	0.397	0.985	0.255	0.312	0.448
BPS MP80	0.173	0.629	0.212	0.583	0.145	0.195
BLL SC596	0.501	0.079	0.248	0.048*	0.004**	0.495

Supplementary Table 6. Count of arabino- and/or xylo-lytic *Bifidobacterium* domains annotated by dbCAN3. Counts are subset by the biochemically characterized activities of their GH subfamily(s') membership.

Strain	Subfamily has α-/β-Ara*f*/*p*ase	GH51	GH43 subfamily has AXOS α-Ara*f*ase	GH43 subfamily has only AG or AN α-L-Ara*f*ase
B. longum subsp. *longum* SC596	21	6	5	9
B. pseudocatenulatum SC585	15	3	4	8
B. pseudocatenulatum MP80	11	3	3	6
B. longum subsp. *longum* SC215	13	6	4	5
B. longum subsp. *longum* APC1473	12	6	4	5
B. longum subsp. *longum* JCM1217	15	5	4	5
B. longum subsp. *longum* SC664	15	3	4	5
B. longum subsp. *longum* APC1480	12	5	2	6
B. catenulatum subsp. *kashiwanohense* JCM15439	10	3	3	4
B. adolescentis ATCC 15703	7	3	2	4
B. animalis subsp. *lactis* ATCC 27536	3	1	2	0
B. bifidum ATCC 29521	1	2	0	1
B. bifidum SC555	1	2	0	1
B. longum subsp. *infantis* ATCC 15697	1	2	0	1
B. breve SC95	2	2	0	1

Supplementary Figure 1. Stacked bar chart of 16S rDNA amplicon sequence ASVs in fecal fermentations from SBP and SBO. Panels; 0 hours, 24 hours and 48 hours of fermentation. X axis sample code; donor #_carbohydrate_replicate. "d", donor; "P", sugar beet pulp; "O", sugar beet oligosaccharide; and fermentation technical replicates "A", "B" and "C".

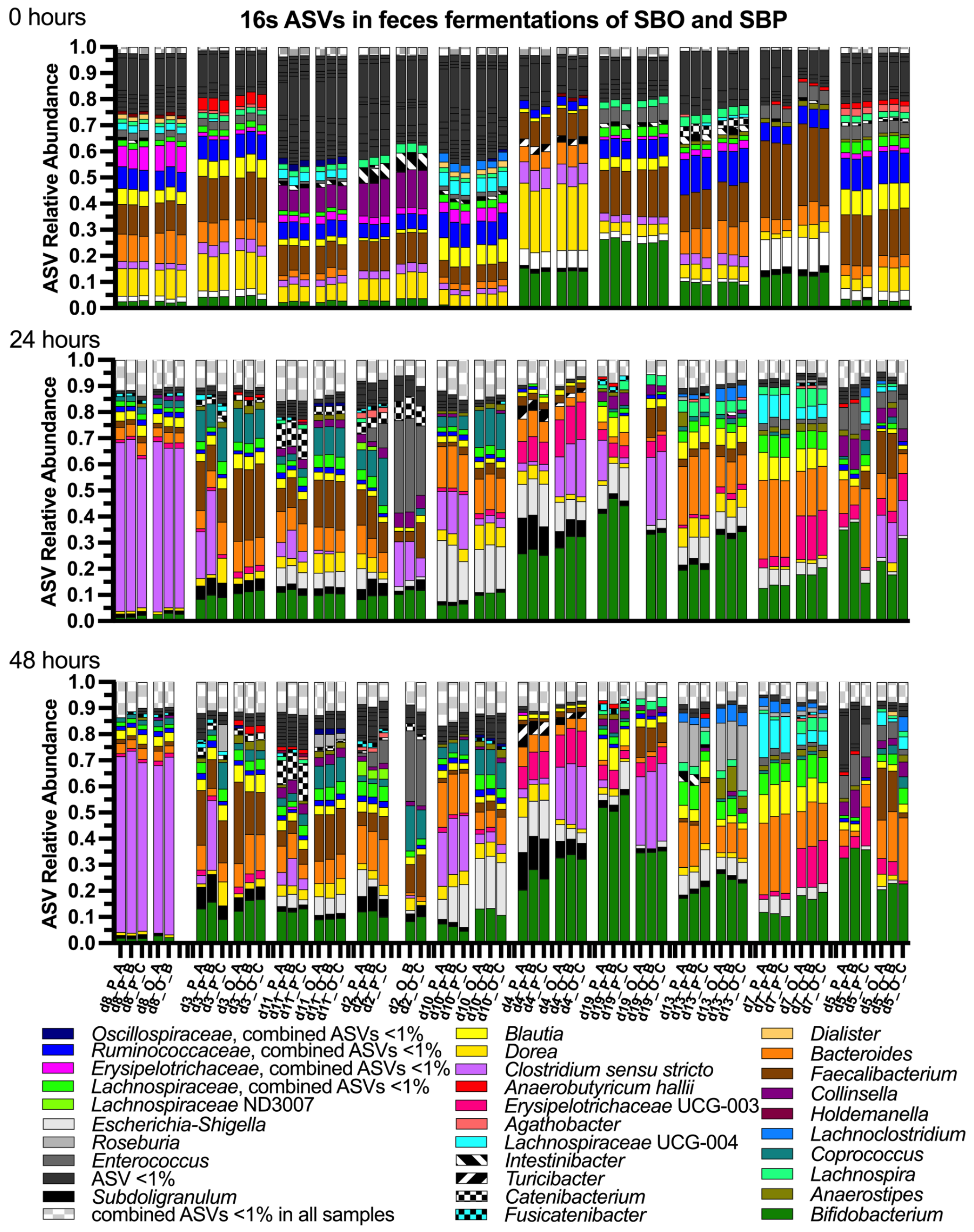

Supplementary Figure 2. (A) Aggregate pH profiles for 10 donor fecal fermentations of SBO and SBP. (B) Individual donor pH profiles.

A.

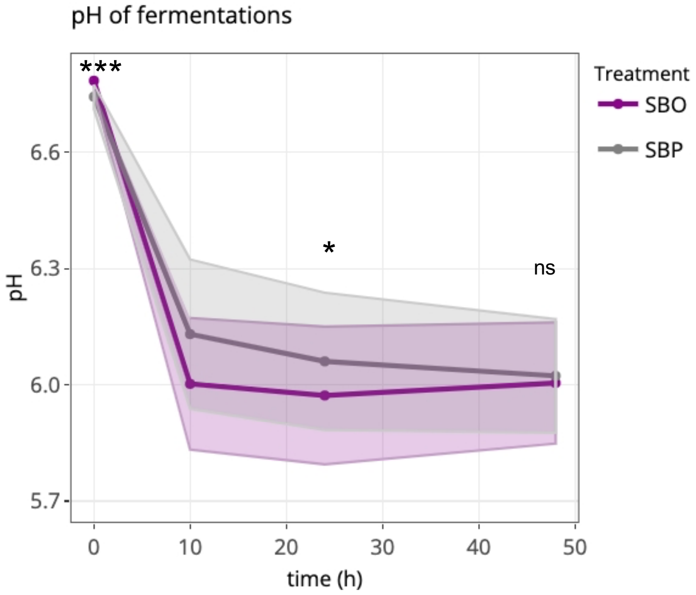

B.

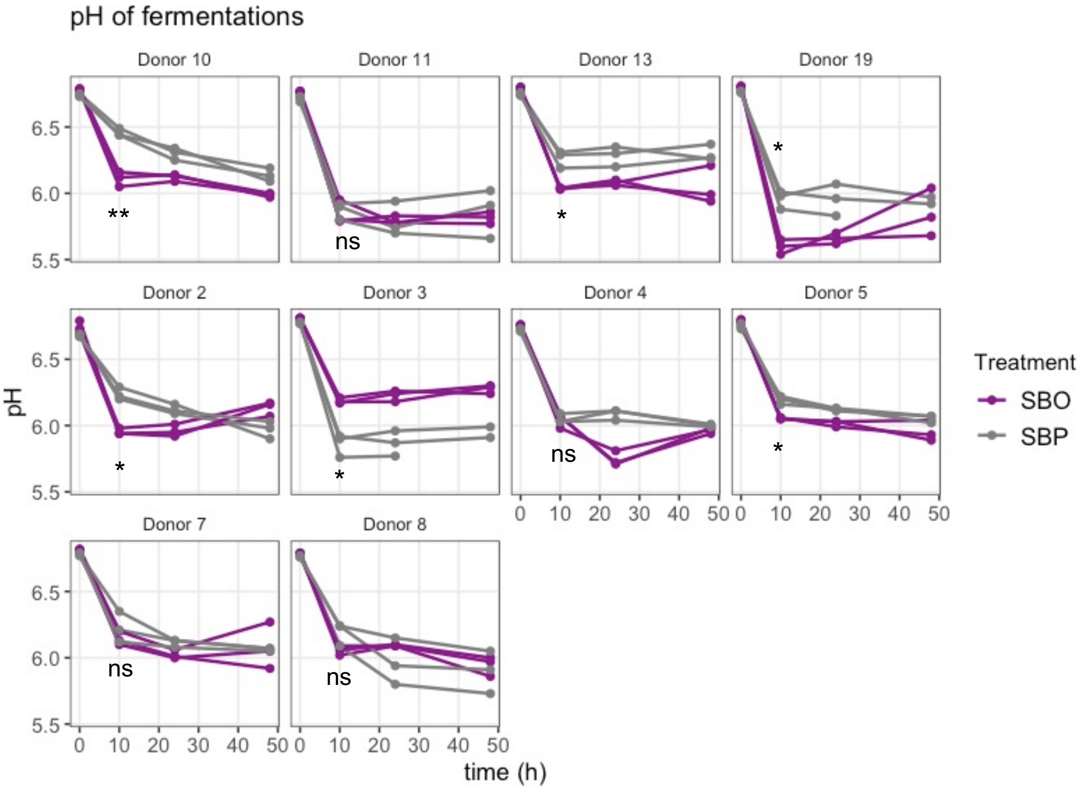

Supplementary Figure 3. Growth of *Bifidobacterium* cultures determined by changes in optical density (OD) overtime. Strains were cultured in duplicate in mMRSC + 1% SBO as a sole carbon source. Species names are abbreviated as follows*: B. pseudocatenulatum* (BPS), *B. bifidum* (BBI), *B. breve* (BBR), *B. animalis* subsp. *lactis* (BAL), B. longum subsp. infantis (BLI). “no inoculum” represents both mMRSC + 1% SBO and mMRSC without carbohydrate. Colored, dotted lines represent inoculated mMRSC without carbohydrate, and each strains’ line is the same color as the mMRSC + 1% SBO.

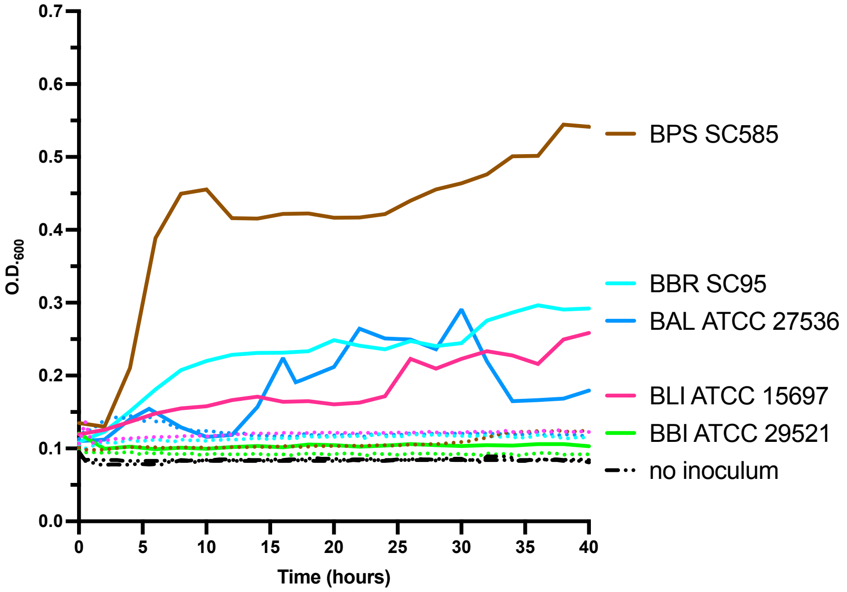

Supplement: Supplemental material — Tables S1 to S6 and Figures S1 to S3. [file aem.02077-24-s0001.pdf]
